# Molecular and Cellular Mechanisms of Respiratory Syncytial Viral Infection: Its Implications for Prophylactic and Therapeutic Pharmaceuticals

**DOI:** 10.1002/mco2.70403

**Published:** 2025-09-29

**Authors:** Xiaowen Liang, Yue Yin, Yuanlong Lin, Shiman Chen, Qi Qian, Jing Yuan, Liuqing Yang, Yang Yang

**Affiliations:** ^1^ School of Pharmacy Shenzhen University Medical School Shenzhen University Shenzhen Guangdong China; ^2^ Shenzhen Key Laboratory of Pathogen and Immunity Shenzhen Third People's Hospital Second Affiliated Hospital School of Medicine Southern University of Science and Technology Shenzhen China

**Keywords:** pathogenic mechanisms, prophylactic strategies, respiratory syncytial virus, therapeutic strategies

## Abstract

Respiratory syncytial virus (RSV) is a notorious pathogen that serves as the leading cause of lower respiratory tract infections (LRTI) among infants, the elderly, and immunocompromised individuals. Its widespread prevalence exerts a considerable burden on global healthcare systems and economies, owing to the high rates of hospitalization and the potential for long‐term health complications. Significant progress has been achieved in RSV prevention strategies during recent years, with three vaccines currently approved worldwide for active immunization in adults aged 60 years and older, as well as pregnant women. Furthermore, the monoclonal antibody nirsevimab has been approved for the prevention of RSV infections in infants. However, effective antiviral treatments for postinfection cases remain an unmet clinical need. This review comprehensively elaborates the molecular and cellular mechanisms of RSV infection, including viral structure, replication cycle, and pathogenic mechanisms. Meanwhile, we systematically summarize the latest advances in preventive and therapeutic agents and analyze the practical applications and existing limitations of current immunization strategies. Furthermore, we discuss and propose the challenges and future directions in drug development. The review provide insights for developing novel and effective prevention and treatment strategies against RSV infection.

## Graphical abstract

Respiratory syncytial virus (RSV) causes substantial global morbidity, particularly in infants and elderly populations. This review summarizes key viral pathogenesis mechanisms and highlights recent advances in both prophylactic (vaccines, antibodies) and therapeutic (antiviral agents) strategies against RSV. Furthermore, the authors discuss and propose current challenges and future directions in RSV drug development, providing insights for research and clinical practice. HSPG, heparan sulfate proteoglycan; IGF1R, insulin‐like growth factor 1 receptor; CXCL4, chemokine ligand 4.

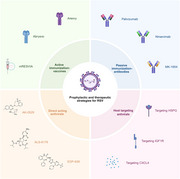

## 1 Introduction

Respiratory syncytial virus (RSV) was first isolated in 1955 by Blount and its colleagues from chimpanzees with respiratory disease [1]. Subsequently, in 1957, Chanock's team was the first to isolate human RSV strains from infants with severe lower respiratory tract infections (LRTI) [2]. Since then, RSV has become a major pathogen causing acute LRTI in infants worldwide, with approximately 33 million cases and 101,400 deaths annually due to their immature immune systems [3]. In developing countries, RSV‐related infant mortality is second only to malaria, representing a significant public health concern [4]. Additionally, the elderly individuals and immunocompromised patients constitute high‐risk groups for severe complications and mortality resulting from RSV infection, as they are more susceptible to developing severe LRTI [5].

RSV stands as the only human‐infecting member of the genus *Orthopneumovirus* within the family *Pneumoviridae*. It boasts a single‐stranded negative‐sense RNA genome that encodes 11 viral proteins [6]. RSV is primarily transmitted via droplets, contaminated surfaces, or direct contact. In infants, RSV is a common cause of bronchiolitis and pneumonia. Typical symptoms encompass wheezing, dyspnea, and pulmonary crackles. In severe cases, these symptoms can escalate to acute respiratory distress [7, 8]. For elderly individuals, RSV infection increases the risk of severe diseases such as pneumonia and congestive heart failure [9]. Compared with other respiratory viruses, RSV exhibits an unusually high reinfection rate, attributed to the inability of natural infection to induce long‐lasting protective immunity: transient mucosal immunity, inadequate maternal antibody transfer, and weak T‐cell memory [10]. Nevertheless, with repeated infections, the cumulative immune responses can mitigate symptoms in subsequent infections, resulting in the typically mild upper respiratory symptoms upon reinfection in older children and adults [10, 11].

Currently, the strategies available for addressing RSV infection are predominantly focused on preventive measures, including vaccination and passive immunization [12]. To date, United States Food and Drug Administration (US FDA) has approved two prophylactic antibodies (palivizumab and nirsevimab) and three RSV vaccines (Arexvy, Abrysvo, and mRESVIA) [13, 14]. Arexvy, which consists of the RSV fusion pre‐fusion (pre‐F) glycoprotein (RSVPreF3) combined with the adjuvant AS01E, has been approved for the prevention of RSV‐related lower respiratory tract disease (LRTD) in individuals aged 60 years and older [13, 15]. Abrysvo is a nonadjuvanted bivalent subunit vaccine authorized for active immunization in pregnant women between 32 and 36 weeks of gestation to safeguard infants under 6 months of age against RSV LRTD [13, 16]. MRESVIA is the first US FDA‐approved mRNA vaccine for RSV prevention in adults ≥60 years [13]. Notably, treatment options for RSV infection remain limited to supportive care, and inhaled ribavirin is the only approved antiviral for RSV treatment [17]. However, due to its nonspecific mechanism, potential genotoxicity, and uncertain efficacy, it is no longer recommended for clinical use [18]. Thus, developing safe and effective antiviral drugs and therapeutic strategies remains an urgent clinical need.

In this review, we summarize recent advances in RSV structural features, pathogenic mechanisms, as well as prevention and treatment strategies. We mainly focused on the structural characteristics of viral proteins and their implications for drug target development, along with current status and challenges in monoclonal antibody (mAb) and small‐molecule inhibitor research.

## 2 Summary of RSV

### 2.1 Global Burden of RSV Infection

RSV is a widely circulating respiratory virus on a global scale and serves as a primary contributor to acute LRTIs [19]. Unlike other respiratory viruses, such as influenza virus and SARS‐CoV‐2, which affect individuals across all age groups, RSV infection exhibits distinct age‐related susceptibility, with the highest disease burden concentrated in infants, the elderly, and immunocompromised individuals [20]. RSV is highly contagious, with almost all children having suffered at least one RSV infection under 2 years of age [21]. A study published in 2022 reported that 33 million RSV‐related acute LRTI occurred globally among children younger than 5 years in 2019, resulting in 3.6 million hospital admissions and 101,400 deaths [3]. Of these, children under 6 months were the age group with the largest proportion of RSV LRTD [3]. The high susceptibility of infants to RSV primarily stems from their extremely narrow and developing airways [22]. Specifically, the diameters of infant bronchiolar lumens are less than half of those in adults [22]. This anatomical characteristic makes infants more prone to airway blockage, which in turn triggers inflammation [23]. Moreover, their immature immune function and inadequate transfer of maternal antibodies further exacerbate the vulnerability to RSV infection [10]. Several known risk factors increase the risk of RSV infection in children, such as congenital or acquired immune disorders, genetic and chromosomal abnormalities disorders, preterm birth, and other underlying respiratory diseases [24]. Such individuals only account for a small proportion of RSV infections, while the highest RSV‐associated mortality rates were found among them [25].

Most older children and adults with RSV infections exhibit symptoms confined to the upper respiratory tract, typically presenting as nonspecific cold symptoms [11]. However, the elderly and frail adults are also at increased risk for severe RSV LRTI [26]. The burden of RSV in older adults is now recognized to be comparable to that of nonpandemic influenza, gaining increasing acknowledgment in recent years [27, 28]. In high‐income countries, an estimated 5.2 million people aged 60 years and above suffered from RSV‐associated illnesses in 2019, resulting in approximately 470,000 hospitalizations and 33,000 deaths [29]. Moreover, older adults with underlying conditions such as chronic lung diseases or cardiovascular diseases face an even higher risk of RSV infection and are more likely to develop severe complications [9, 30, 31]. This increased susceptibility may be attributed to age‐related pulmonary physiological changes, immune senescence, and reduced levels of RSV‐specific serum immunoglobulin (Ig) and nasal IgA [24, 30, 32].

RSV infection in immunocompromised individuals can significantly increase the risk of persistent wheezing and asthma, which may be associated with exacerbated Th2 immune bias [33]. Immunocompromised patients have a significantly higher mortality rate from RSV infection than the general population, especially hematopoietic stem cell transplant (HSCT) recipients, solid organ transplant recipients, and lung transplant (LTx) recipients (LTRs) [34]. Up to 50% of RSV infections among these individuals progress from upper respiratory tract infections (URTI) to LRTI, and the related mortality rate approaches 80% [35].

RSV epidemics display distinct seasonal and geographical patterns [36]. In temperate regions, RSV outbreaks generally occur during winter month. Specifically, the Northern Hemisphere typically witnesses a well‐defined epidemic peak from November to March, while the Southern Hemisphere experiences a high prevalence of RSV between June and September, which is closely associated with low‐temperature and low‐humidity environmental conditions [37, 38, 39]. In contrast, tropical regions, such as Southeast Asia and African countries, demonstrate a year‐round low‐level circulation with only slight increases during rainy seasons [38, 39]. Consequently, region‐specific prevention strategies should be implemented. In temperate zones, targeted measures such as maternal vaccination and mAb prophylaxis for high‐risk infants should be initiated prior to the epidemic season, whereas tropical regions require year‐round surveillance and immunization programs [40]. Notably, nonpharmaceutical interventions implemented during the COVID‐19 pandemic—such as reduced social contact and mask‐wearing—not only decreased SARS‐CoV‐2 transmission but also significantly reduced exposure opportunities to other respiratory viruses like RSV [41, 42, 43]. The resulting “immunity debt” contributed to abnormally high RSV infection and hospitalization rates in multiple regions worldwide following the relaxation of containment measures against COVID‐19 [44, 45, 46]. Moreover, following the COVID‐19 pandemic, the RSV epidemic season underwent significant changes. For instance, an unusual peak occurred in the summer (June–August) of 2021 in the United States, arriving 3–5 months earlier than previous years [44, 47]. Current global trends indicate that while RSV activity is gradually normalizing seasonally, some regions still experience fluctuations [48]. This highlights the need to establish a more flexible surveillance system, closely monitor the onset and peak timing of future epidemics, and promptly adjust prevention and control strategies.

### 2.2 RSV Genome and Structure

RSV is an enveloped, negative‐sense, single‐stranded RNA virus belonging to the Paramyxoviridae family, and it contains three morphologies: spherical, asymmetric, and filamentous [49, 50]. The Filamentous form, with a diameter of 0.5–12 µm and a length of 1–10 µm, is the dominant form [51]. Based on antigenic reactivity to mAbs, RSV is categorized into two subtypes, A and B, which are further subdivided into 11 RSV‐A genotypes and 23 RSV‐B genotypes [52]. Both subtypes can be transmitted simultaneously during an outbreak. However, only one subtype is predominant, and periodic genotype substitution exists commonly [53, 54].

**FIGURE 1:**
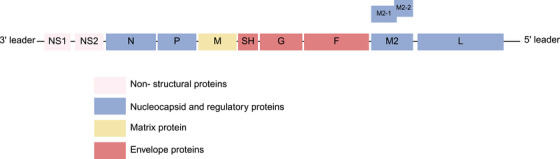
The genome structure of RSV. F, fusion protein; G, adhesion protein; L, large polymerase protein; M, matrix protein; M2‐1 protein, transcription factor; N, nucleoprotein; NS1, nonstructural protein 1; NS2, nonstructural protein 2; P, phosphoprotein; SH, small hydrophobic protein.

The RSV viral genome is 15.2 kb in length and contains 10 genes in the order 3′‐NS1‐NS2‐N‐P‐M‐SH‐G‐F‐(M2‐1/M2‐2)‐L‐5′, encoding 11 proteins (Figure 1) [6]. The nonstructural proteins [NS1] and [NS2] help to evade the innate immune response. They inhibit apoptosis and interferon (IFN)‐activated signaling pathways, thus promoting viral replication [55, 56]. The small hydrophobic proteins [SH], adhesion proteins [G], and fusion proteins [F] are transmembrane glycoproteins. Among them, F and G proteins are crucial for viral invasion of the host cell. The F protein, typically in trimeric form, transitions from a pre‐F to a post‐fusion (post‐F) conformation during the fusion of the virus and host cell membrane [57]. F protein is as highly conserved across different strains, making it the primary target for RSV vaccine development [58]. The small hydrophobic proteins [SH] function as viral channel proteins, enhancing membrane permeability and regulating host cell apoptosis [59]. The nucleoprotein [N] wraps the viral genome to protect it from degradation and serves as a template for transcription and replication [60]. The large polymerase protein [L], nucleoprotein [N], and phosphoprotein [P] form the RNA‐dependent RNA polymerase (RdRp) complex, essential for viral transcription and replication. Four nucleocapsid proteins (N, P, L, and M2‐1) interact with viral genomic RNA assembly to form a helical ribonucleoprotein (RNP) complex [61]. The M2 gene has two overlapping open reading frames encoding two proteins, M2‐1 and M2‐2. M2‐1 connects M protein to RNP, while M2‐2 is involved in transcription and replication processes [62]. The matrix protein[M], located inside of the viral envelope, is necessary for the assembly and release of viral particles [63].

### 2.3 The Life Cycle of RSV

**FIGURE 2:**
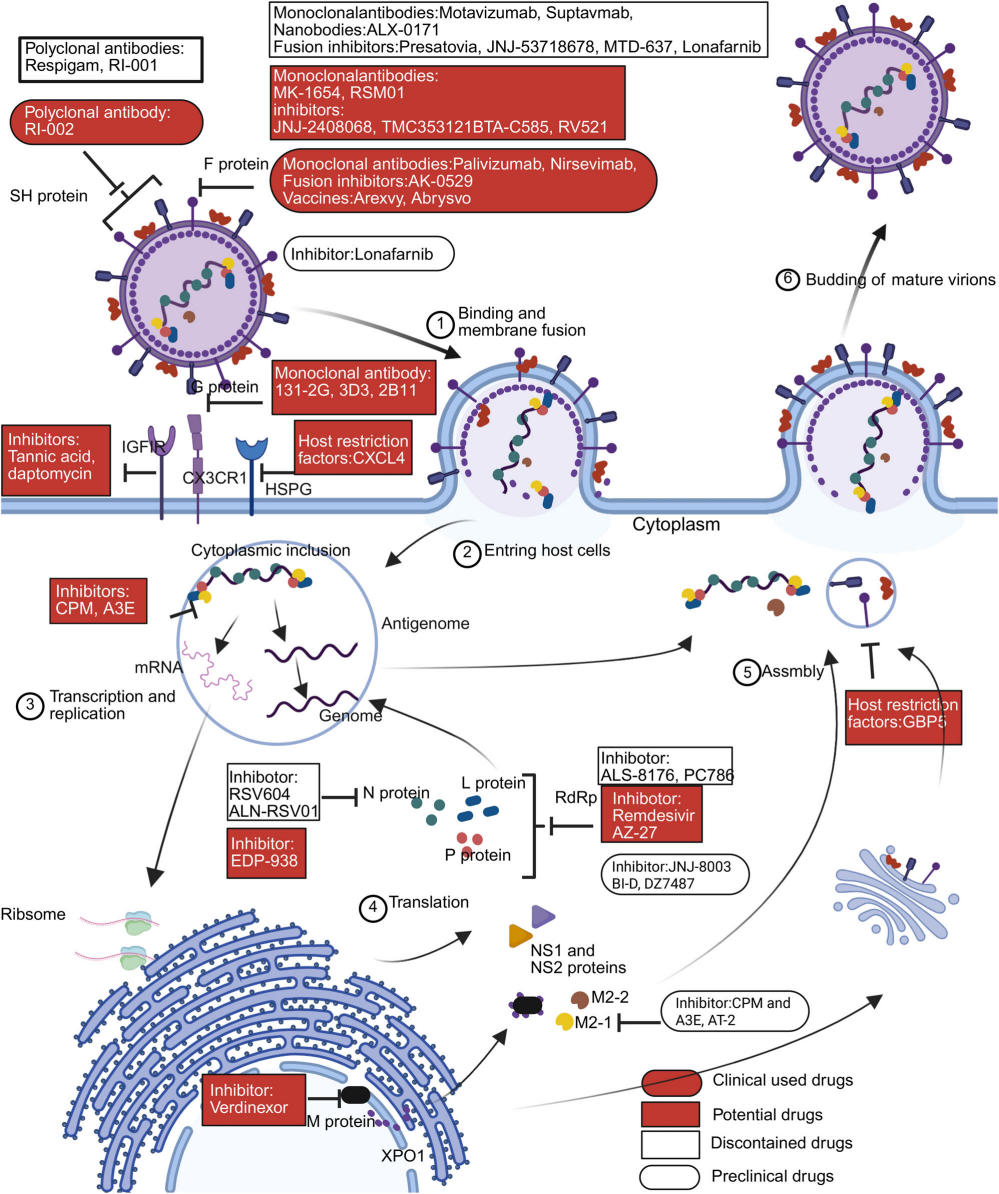
The life cycle of RSV in hosts and targets for anti‐RSV drugs. The complete life cycle of RSV infection: RSV adheres to the apical surface of ciliated epithelial cells via G protein, then fuses with the host cell membrane mediated by F protein, releasing nucleocapsid into the host cell cytoplasm. After entering the cell, the virus replicates and transcribes in the cytoplasm of the inclusion bodies. RdRp is responsible for transcribing the viral genome into mRNA and synthesizing positive‐sense antigenomic intermediates for replication of the new antisense genome. The M2‐2 protein mediates the switch from viral transcription to replication. The newly replicated viral genome is encapsidated by the N protein, which then binds to the P, L, and M2‐1 proteins to form RNP. Meanwhile, the viral envelope glycoproteins undergo endoplasmic reticulum and Golgi apparatus modifications and subsequently, interact with RNP in coordination with M proteins. Eventually, they are assembled into spherical or filamentous mature polymorphic viral particles and released by budding from the host cell membrane. Current drugs and therapy targets are shown. CX3CR1, CX3C motif chemokine receptor 1; CXCL4, chemokine ligand 4; GBP5, guanylate binding protein 5; HSPG, heparan sulfate proteoglycan; IGF1R, insulin‐like growth factor 1 receptor; RdRp, RNA‐dependent RNA polymerase.

RSV primarily targets airway epithelial cells in the host [64, 65]. The replication cycle of RSV involves multiple distinct stages, with each stage being mediated by specific viral proteins in coordination with host cellular machinery. A comprehensive understanding of the viral life cycle provides crucial theoretical foundations for developing targeted antiviral strategies (Figure 2).

#### 2.3.1 Viral Attachment and Entry

RSV infection initiates with viral particle attachment to the apical surface of ciliated epithelial cells. The RSV G protein recognizes and binds to specific receptors on the host cell surface, such as nucleolin, CX3C motif chemokine receptor 1 (CX3CR1), and heparan sulfate (HS) proteoglycan (HSPG) [66, 67, 68]. Following successful attachment, the F protein undergoes conformational changes that drive the fusion between the viral envelope and host cell membrane, resulting in the release of the nucleocapsid into the host cell's cytoplasm [69]. Interestingly, although the G protein facilitates this process, it is not absolutely required for viral entry, as the F protein can independently interact with immobilized heparin or cellular heparin sulfate to mediate attachment, albeit with reduced efficiency compared with G protein‐mediated binding [70, 71].

Besides membrane fusion, RSV can enter host cells through macropinocytosis, a process dependent on actin remodeling and Na⁺/H⁺ exchangers [72]. The G protein binds to specific receptors on the host cell membrane, triggering a downstream signaling cascade mediated by the Cdc42–PAK1 axis. This induces extensive cytoskeletal reorganization, leading to the formation of characteristic membrane ruffles and protrusions at the viral attachment site. The viral particles are subsequently enveloped by these membrane extensions and internalized into the cell via macropinocytosis. The newly formed macropinosomes exhibit Rab5^+^ markers and undergo gradual acidification, creating a favorable environment for viral fusion [72, 73, 74]. Ultimately, the F protein mediates the merging of the viral envelope with the macropinosome membrane, releasing the viral nucleocapsid into the cytoplasm [72].

#### 2.3.2 Viral Transcription and Genome Replication

Upon entering the host cell, viral transcription and replication occur within cytoplasmic inclusion bodies (IBs) [75]. This process depends on the RSV polymerase and the host cell's translation system. The RSV polymerase is a multifunctional RdRp composed of the L and the P proteins. The RdRp serves a dual function which both transcribes the viral genome into mRNA and synthesizes positive‐sense antigenomic intermediates for the replication of new antisense genome [76]. The transcription process initiates when the RdRp recognizes the promoter region at the 3′ terminus of the viral RNA, and then sequentially transcribes each viral gene from their respective gene start (GS) sequences and terminating at gene end (GE) sequences [77, 78]. The M2‐1 protein plays a critical role during transcription by preventing premature termination and ensuring complete mRNA synthesis [79]. Newly synthesized mRNAs undergo 5′ capping and 3′ polyadenylation mediated by the L protein, after which they are recognized by the host cell's ribosome complex for viral protein translation [80, 81].

With the accumulation of viral N protein to sufficient levels and the help of M2‐2 protein, the function of RdRp switches from transcription to genome replication [82]. During this process, the RdRp overcomes GS/GE signals to continuously synthesize full‐length antigenomic RNA [83]. Both newly replicated antigenomes and genomic RNAs are encapsidated by N protein, then associate with P, L, and M2‐1 proteins to form helical RNPs that serve as templates for subsequent rounds of RNA synthesis [84, 85].

#### 2.3.3 Viral Assembly and Budding

The M protein plays a crucial role in viral assembly and budding [86]. During the late stage of viral replication, M proteins located in the nucleus are recruited to cytoplasmic IBs, where they interact with preformed RNPs. Simultaneously, M proteins are also recruited to lipid rafts to form glycoprotein complexes with viral envelope glycoproteins (G, F, and SH proteins) that have undergone posttranslational modifications in the endoplasmic reticulum and Golgi apparatus [69, 87]. Under the orchestration of M proteins, these glycoprotein complexes assemble with RNPs carrying the viral RNA genome to form polymorphic mature virions exhibiting either spherical or filamentous morphology, which are ultimately released through host cell membrane budding [63, 88]. Notably, the viral surface F protein not only mediates virus–host cell fusion but also promotes fusion between infected cells and neighboring uninfected cells, leading to the formation of multinucleated giant cells (syncytia) [89].

### 2.4 Mechanisms of RSV Pathology

**FIGURE 3:**
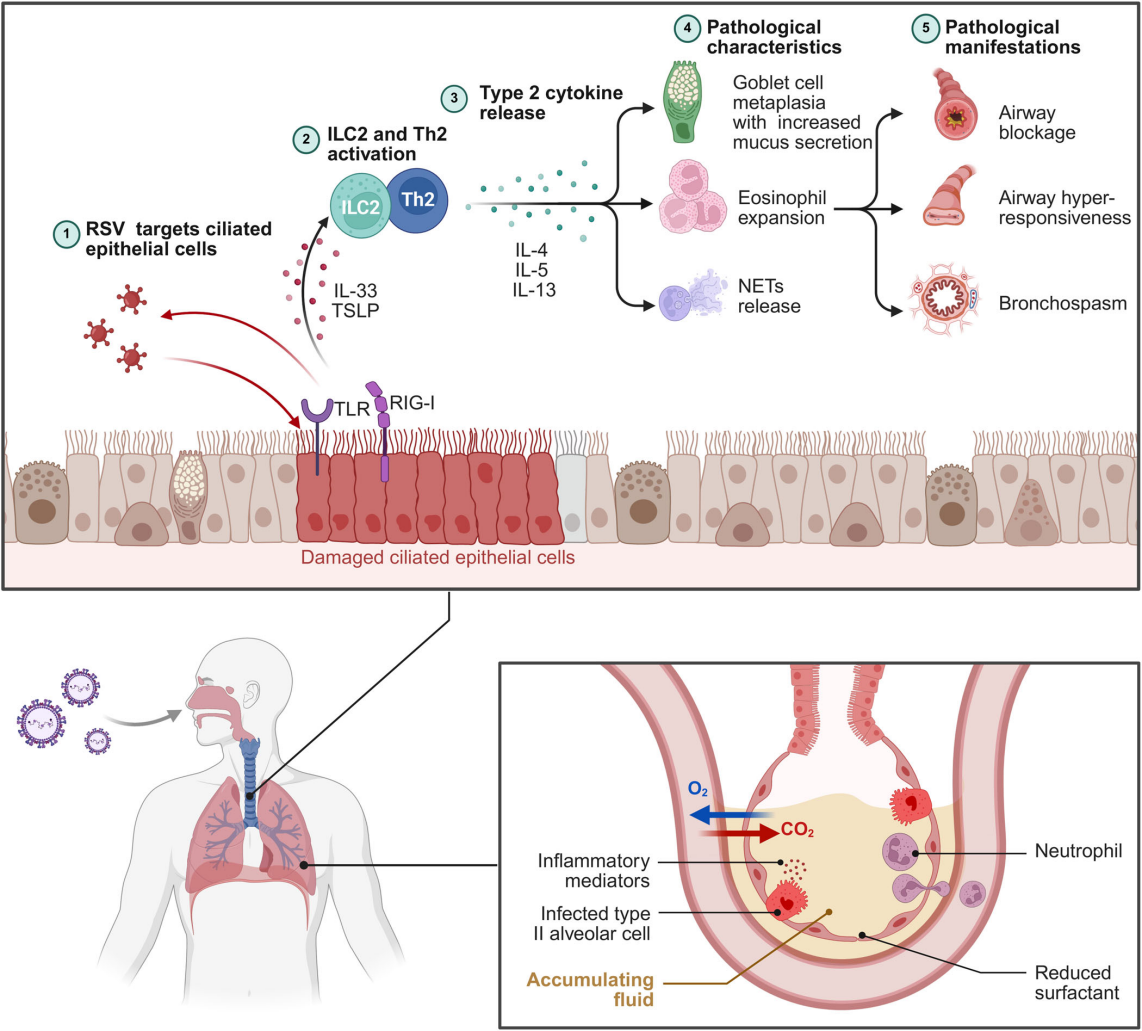
Mechanisms of RSV pathology. RSV first invades the upper respiratory tract and progressively spreads to the lower airways. The virus primarily targets ciliated epithelial cells, causing cellular damage and massive shedding. Concurrently, viral recognition by host pattern recognition receptors (TLRs and RIG‐I) activates immune responses and induces the release of IL‐33 and TSLP, which then trigger ILC2 and Th2 cell activation with excessive type 2 cytokine secretion. The exaggerated inflammatory response promotes immune cell recruitment and characteristic pathological changes, leading to bronchial obstructive pathology. Upon alveolar invasion, RSV suppresses surfactant production and secretion by type II alveolar epithelial cells, impairs alveolar fluid clearance, and induces inflammatory vascular leakage. These pathological alterations collectively result in alveolar collapse, pulmonary edema as well as gas exchange impairment, and ultimately manifest as severe pneumonia. ILC2, type 2 innate lymphoid cell; NETs, neutrophil extracellular traps; RIG‐I, retinoic acid‐inducible gene I; TLR, toll‐like receptor; TSLP, thymic stromal lymphopoietin.

The pathogenesis of RSV is closely related to the host's excessive immune response (Figure 3) [69]. After transmission via droplets or contact, the virus initially infects the epithelial cells of the nasopharynx and larynx, causing upper respiratory symptoms such as nasal congestion, rhinorrhea, and sore throat [7]. Subsequently, the virus spreads to the lower respiratory tract via ciliary movement and the cough reflex, invading the terminal bronchioles and alveoli. In ciliated epithelial cells, RSV disrupts the mucociliary clearance system, impairing ciliary movement and causing epithelial cell shedding [90]. Simultaneously, the virus is recognized by host pattern recognition receptors such as toll‐like receptors (TLRs) and retinoic acid‐inducible gene I (RIG‐I), activating immune responses and triggering the release of various cytokines and chemokines, including IL‐33 and thymic stromal lymphopoietin (TSLP) [65, 91, 92, 93]. IL‐33 activates type 2 innate lymphoid cells (ILC2s) and Th2 cells, promoting the excessive secretion of type 2 cytokines such as IL‐4, IL‐5, and IL‐13, thereby driving a Th2‐biased immune response [94, 95, 96]. This aberrant immune polarization leads to a series of characteristic pathological changes including goblet cell hyperplasia and metaplasia with markedly increased mucus secretion and massive eosinophil infiltration at inflammatory sites. Meanwhile, neutrophils are also recruited to the infected area, participating in antiviral defense through the release of neutrophil extracellular traps (NETs) [92, 97, 98, 99]. However, excessive inflammatory responses cause NETs to mix with shed ciliated epithelial cells and excess mucus, forming dense mucus plugs that lead to airway obstruction [98, 100]. Additionally, the inflammatory mediators released during infection enhance smooth muscle contractile responsiveness, leading to airway hyperreactivity and bronchospasm [101, 102]. This constitutes a key pathological basis for the clinical manifestations of wheezing and dyspnea, and frequently triggers bronchiolitis in infants [101].

Furthermore, if the virus breaches the terminal bronchioles and invades the alveoli, it can trigger severe pneumonia symptoms [103, 104]. Viral replication within alveoli suppresses the synthesis and secretion of pulmonary surfactant by type II alveolar epithelial cells, leading to increased alveolar surface tension and compromised alveolar stability. Concurrently, activation of the protein kinase C signaling pathway inhibits epithelial sodium channel function, significantly impairing active alveolar fluid clearance and leading to intra‐alveolar fluid accumulation [105, 106]. The accompanying inflammatory response promotes massive release of inflammatory mediators such as TNF‐α and VEGF, resulting in disruption of the alveolar–capillary barrier integrity and markedly increased vascular permeability [107]. These pathological alterations collectively induce alveolar collapse and pulmonary edema formation, which severely compromise gas exchange function and ultimately manifests as severe clinical symptoms including hypoxemia and respiratory distress [108].

Notably, RSV infection significantly increases the risk of secondary bacterial infections [109]. The virus upregulates the expression of bacterial receptors on the surface of respiratory epithelial cells, including intercellular adhesion molecule 1 (ICAM‐1), platelet‐activating factor receptor, and carcinoembryonic antigen‐related cell adhesion molecule 1 [24, 109]. Concurrently, RSV‐induced impairment of mucociliary clearance compromises bacterial elimination from the lower airways [24, 109]. These alterations create favorable conditions for colonization and invasion by bacterial pathogens such as *Streptococcus pneumoniae* and *Haemophilus influenzae* in the lower respiratory tract [110, 111]. Clinical data indicate that children with RSV infection complicated by bacterial coinfection often experience more severe disease and face greater treatment challenges [112, 113].

In the long term, severe RSV infection in early childhood is significantly associated with an increased risk of asthma and recurrent wheezing [8]. This may be due to the establishment of abnormal immune memory during viral infection, particularly the persistent activation of Th2 immune responses, leading to chronic airway inflammation and remodeling [33, 114, 115]. Additionally, severe infections during critical periods of lung development may interfere with normal alveolarization, leaving some children with persistent pulmonary dysfunction [115]. Therefore, early identification of high‐risk children and targeted preventive measures are crucial.

## 3 RSV Preventive and Therapeutic Antibodies

Vaccines for the elderly and pregnant women have been successively approved for market use, providing effective active immunization protection for high‐risk populations [14]. However, there are no vaccines are currently approved for infants and young children, leaving passive immunization with antibodies as the sole preventive/therapeutic option against RSV in this vulnerable population. This section introduces RSV‐related antibodies, including those already available in the market, those currently in the development pipeline, and those been discontinued.

### 3.1 RSV Ig

Passive immunity refers to the specific immune protection acquired by an organism through passive reception of antibodies, sensitized lymphocytes or their derivatives [14]. RSV Ig is a polyclonal antibody preparation extracted from human plasma, boasting high‐titer RSV‐neutralizing antibodies. As early as 1984, passive immunotherapy using high‐titer RSV Ig intravenous (IGIV) was demonstrated to be feasible in cotton rat and owl monkey models [116, 117].

#### 3.1.1 Respigam (RSV‐IGIV)

Respigam is the first US FDA‐approved immunoprophylactic agent with high titers of neutralizing antibodies against RSV, for the prevention of severe RSV bronchiolitis in children in 1993 [118]. It has been shown that treatment with high dose of Respigam (750 mg/kg) significantly reduced the frequency of hospitalization, total days of hospitalization, and days in intensive care unit (ICU) in 249 RSV infected children with congenital heart disease (CHD), bronchopulmonary dysplasia (BPD) or in preterm infants [119]. Although six children died during the trial period, none of the deaths were attributed to diseases caused by IGIV use or RSV infection [119]. Another two studies have evaluated the safety and efficacy of Respigam in the treatment of high‐risk infants: a prevention study and a cardiac study. In the prevention study, monthly administration of Respigam was safe and well tolerated in infants born prematurely and children with BPD, and effectively reduced the length of hospital stay [120]. In cardiac study, Respigam treatment paradoxically increased the incidence of unexpected cyanotic episodes and poor postoperative outcomes in children with CHD, probably due to IGIV‐induced hyperviscosity and relative erythrocytosis [121]. Given these considerations, Respigam is approved for use in premature infants and children with BPD, not the children with CHD. However, Respigam has some limitations, including high cost, high preparation requirements, and need for intravenous administration. Respigam was withdrawn from the market in 2004, following the US FDA approval of the first anti‐RSV mAb palivizumab in 1998 [122].

#### 3.1.2 RI‐001

People with primary immunodeficiency disease (PIDD) cannot often produce sufficient antibodies to combat infections [123]. Regular administration of Ig to replenish antibodies may reduce the risk of serious bacterial or viral infections in these patients [124, 125]. RI‐001 is an intravenous Ig preparation derived from a mixed plasma pool of healthy adult donors, containing higher neutralizing anti‐RSV titers (>1.5 times the mean of commercial IGIV) than RespiGam [126]. A phase II trial involving 15 immunocompromised RSV‐infected patients demonstrated a significant increase in RSV‐neutralizing antibody titers in serum with excellent tolerance [127]. Although four deaths occurred, three of these patients were at close to 100% mortality before receiving the drug, and no deaths were related to administrations of R1‐001 [127]. RI‐001 is considered a promising drug candidate for the treatment of immunocompromised or immunodeficient RSV‐infected patients. Nevertheless, the clinical use of RI‐001 is limited, as it was subject to the same limitations as RespiGam in PIDD and other patients who derive benefits from IG use [126].

#### 3.1.3 RI‐002

RI‐002 is derived from a minimum of 1000 normal source donors, thus meeting US FDA guidelines for treating patients with PIDD. Meanwhile, RI‐002 meets the minimum titer requirement for measles, polio, tetanus, and hepatitis B, and also contains standardized elevated levels of RSV‐neutralizing antibodies [126]. The efficacy and safety of RI‐002 in patients with PIDD have been evaluated in an open‐label, phase III study conducted in the United States. The primary efficacy endpoint was the annual incidence of acute serious bacterial infection (SBI), with no SBI observed over 55.88 study years (SBI incidence below the criterion of <1.0 SBI per subject per year). The secondary efficacy endpoints also showed low rates of all types of events, including days missed from work due to infection (1.66 days per subject per year), unscheduled physician visits (0.97 per subject per year), and days hospitalized due to infection (0.018 hospitalizations per subject per year). A pharmacokinetic study revealed that RI‐002 maintained IgG and specific antibody levels similar to baseline, with little variation in antibody levels across different doses and dosing cycles. Two serious adverse events were reported during the study but were not related to RI‐002. These data demonstrate the significant efficacy and favorable safety profile of RI‐002 in patients with PIDD [128].

Excessive viral replication may drive RSV pathogenesis in patients with PIDD [129, 130]. Boukhvalova et al. [131] reported that RI‐002 inhibited prolonged replication of RSV in immunocompromised cotton rats, accelerated viral clearance, and reduced damage to pulmonary tissue and airway lining. In addition, RI‐002 has high antibody potency against RSV and significantly increased specific antibodies against other respiratory viruses such as influenza A and B, parainfluenza viruses, human metapneumovirus, and coronavirus [132]. These respiratory viruses are very common in patients with PIDD and often lead to bacterial superinfections [133]. RI‐002 is currently approved for the treatment of primary humoral immunodeficiency disease in adults and adolescents under the trademark Asceniv.

### 3.2 Anti‐F mAbs

mAbs outperform polyclonal antibodies in RSV prevention with high neutralizing potency, extended half‐life, and superior administration convenience, making them the preferred passive immunization choice [134]. The key neutralizing epitopes of RSV are concentrated on the F protein, a type I transmembrane glycoprotein that exists in two conformational states: pre‐F and post‐F [26]. Although the post‐F conformation is more stable, the antibodies it induces exhibit limited neutralizing capacity. In contrast, the metastable pre‐F conformation contains more critical neutralizing epitopes that elicit stronger antibody responses [135]. To date, at least seven antigenic sites have been identified on the F protein, with site‐specific antibodies successfully developed [135, 136]. Among them, sites Ø and V are unique to the pre‐F conformation, while the other sites (I–IV) are present in both the pre‐F and post‐F conformations [137]. Of particular significance, neutralizing antibodies targeting site Ø (e.g., nirsevimab) demonstrate the strongest neutralizing activity. The related epitope is located at the apex of the pre‐F trimer and is formed by the folding of the heptad repeat A (HRA) region into a globular head structure—its distinctive conformational characteristics underlie the highly efficient neutralizing antibody response [138, 139]. However, the relatively higher variability of site Ø across different RSV subtypes has raised concerns about potential antibody escape, highlighting an important consideration for therapeutic development [138, 139].

#### 3.2.1 Palivizumab (Synagis)

**FIGURE 4:**
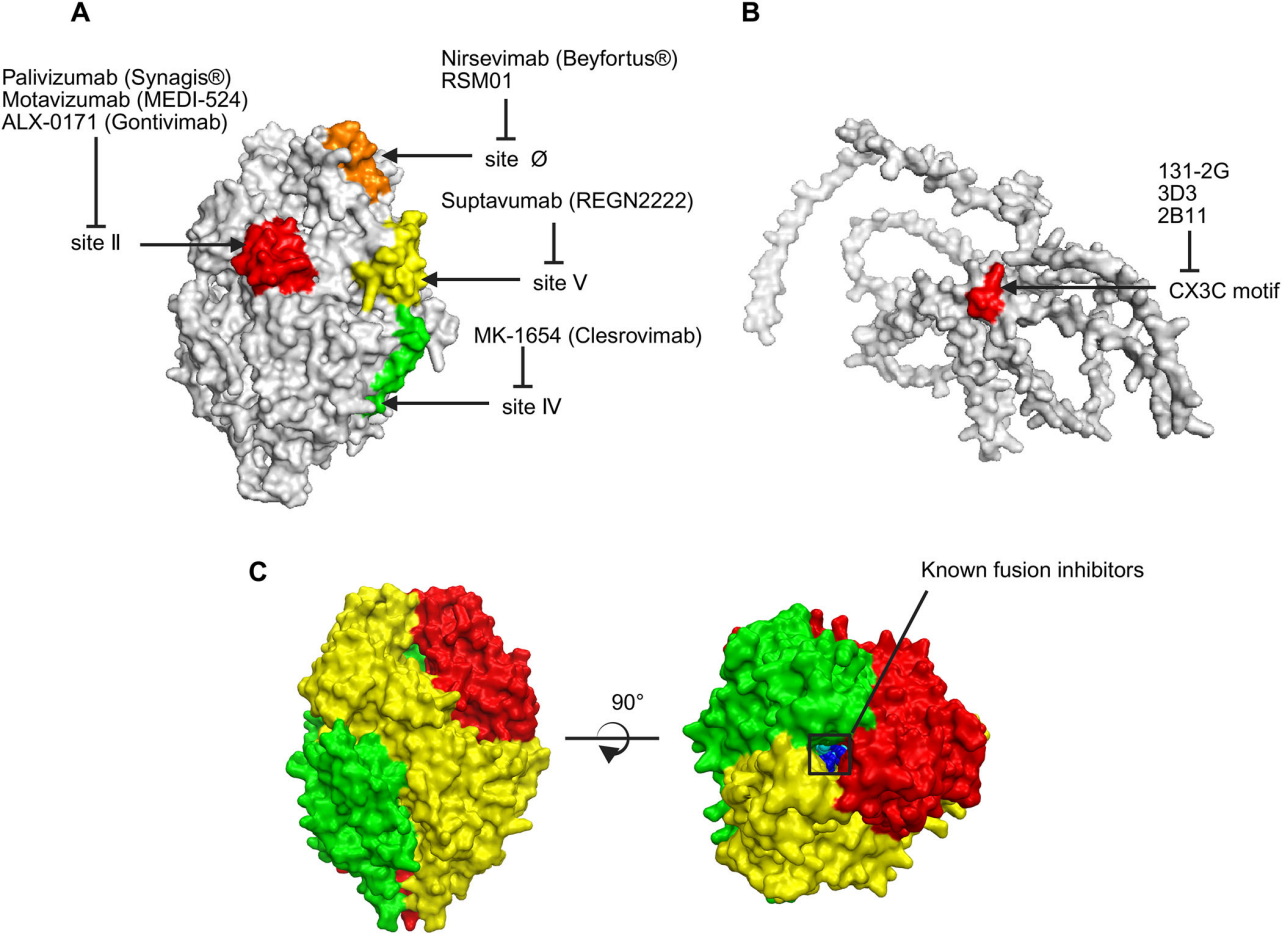
Summary of targeting sites for monoclonal neutralizing antibodies and fusion inhibitors against RSV. (A) Targeting sites of palivizumab (Synagis), motavizumab (MEDI‐524), suptavumab (REGN2222), nirsevimab (Beyfortus), MK‐1654 (clesrovimab), RSM01 and ALX‐0171 (gontivimab) against RSV F protein. (B) Targeting sites of 131–2G, 3D3, and 2B11 against RSV G protein. (C) Known fusion inhibitors targeting the central hydrophobic cavity within the prefusion trimer of RSV F protein.

Palivizumab is a humanized murine mAb targeting antigenic site II of the pre‐F fusion protein (Figure 4A), which is currently approved for preventing RSV in premature infants and those with CHD or BPD [140]. The phase III (IM‐pact‐RSV) study, published in 1998, enrolled 1502 infants born prematurely (≤35 weeks gestation) or with BPD, and palivizumab prophylaxis resulted in a 55% reduction in RSV hospitalizations (4.8 vs. 10.6%) when compared with the placebo group [141]. In high‐risk infants, palivizumab has been proven to reduce morbidity but not mortality [142]. Furthermore, due to its limited cost‐effectiveness, the American Academy of Pediatrics revised the eligibility criteria for palivizumab therapy in 2014 to limit its clinical use. After the revision, routine palivizumab treatment for children without BPD born between 29 and 35 weeks of gestation was eliminated and palivizumab prophylaxis after RSV infection was discontinued [143].

#### 3.2.2 Motavizumab (MEDI‐524)

Motavizumab, a second‐generation humanized recombinant IgG1 mAb, was derived by in vitro affinity maturation of the murine complementary decision regions of the heavy and light chains of palivizumab [144]. As same as palivizumab, it targets antigenic site II of the pre‐F fusion protein (Figure 4A). Whereas it binds to RSV F glycoprotein 70‐fold better than palivizumab and has approximately 20‐fold greater in vitro RSV neutralization properties [144]. This increase in neutralizing activity may be associated with further reduced RSV replication and significant reductions in cytokine and chemokine concentrations [145]. MEDI‐524 also performed better in terms of long‐term pulmonary abnormalities compared with palivizumab. MEDI‐524 significantly reduced RSV viral load in the lung and upper respiratory tract, decreasing the duration and severity of the disease [146].

In a phase I/II trial, motavizumab administered once monthly at 15 mg/kg in high‐risk children demonstrated a favorable safety and tolerability profile, with similar pharmacokinetics to other IgG1 antibodies [147]. In a noninferiority phase III trial in preterm infants, motavizumab recipients showed a 26% reduction in RSV hospitalizations and a 50% reduction in RSV‐specific outpatient medically attended LRTIs (MALRIs) compared with palivizumab recipients, with no significant differences in safety profiles between the two groups [148]. Notably, motavizumab recipients exhibited a relative increase in cutaneous events (19.3 vs. 16.2%), including urticaria, allergic dermatitis, and eyelid edema, which is consistent with possible skin hypersensitivity reactions [149]. Motavizumab was not licensed by the US FDA due to the increased risk of hypersensitivity reactions. Currently, MedImmune has discontinued its development program for the prevention of RSV.

#### 3.2.3 Suptavumab (REGN2222)

Suptavumab is another palivizumab‐based anti‐F protein mAb targeting antigenic site V of the pre‐F fusion protein [150]. Although preclinical studies have demonstrated the high efficiency of suptavumab against RSV in a cotton rat model, suptavumab fails to exhibit clinical benefit regarding RSV‐related inpatient or outpatient LRTI in a phase III clinical trial in preterm infants [151]. The primary reason for this failure was the discovery of 2 amino acid mutations (L172Q and S173L) on all circulating RSV‐B strains, which resulted in the inability of suptavumab to bind to its F protein [151]. Consequently, the development of suptavumab was terminated due to failure to meet clinical endpoints.

#### 3.2.4 Nirsevimab (Beyfortus, MEDI‐8897)

Nirsevimab, codeveloped by AstraZeneca and Sanofi, is a long‐acting recombinant neutralizing human IgG1κ mAb that targets a highly conserved site Ø on the prefusion conformation of the RSV (Figure 4A) [152, 153]. It has been engineered with a triple amino acid substitutions (M252Y/S254T/T256E; YTE) in the Fc domain, which extended its serum half‐life [154].

The safety and efficacy of nirsevimab for preventing RSV LRTI have been evaluated in phase IIb and phase III (MELODY) clinical trials among healthy, late preterm, and term infants [155, 156]. In the phase IIb trial, administration of a single dose of nirsevimab prevented LRTI (70.1% efficacy) and hospitalization (78.4% efficacy) due to RSV in healthy preterm infants (gestation between 29 and <35 weeks), with a good safety profile [155]. The phase III study (MELODY) included 1490 healthy late preterm and term infants (gestational age greater than 35 weeks). The incidence of medically attended RSV‐related LRTI within 150 days (primary endpoint) was 1.2% and the incidence of hospitalization for RSV‐related LRTI (secondary endpoint) was 0.6% in infants given nirsevimab, which was significantly lower than that in infants received placebo (5.0%, 1.6%) [156]. Children from the phase III MELODY trial were subsequently followed up in the second RSV season. No increase in RSV LRTI or enhanced disease severity was observed in nirsevimab recipients compared with placebo recipients [157]. An additional phase II/III palivizumab‐controlled trial (MEDLEY) evaluated the efficacy of nirsevimab in preterm infants and infants with BPD or CHD. The results showed that nirsevimab was similar to palivizumab in terms of tolerability and safety [158]. Based on the phase IIb trial, the phase III MELODY trial, and the phase II/III MEDLEY trial, nirsevimab was approved by the US FDA in 2023 [159]. It is the world's first single‐dose RSV prophylaxis that can be broadly applied to the infant population.

NIRSE‐GAL is a large population‐based follow‐up study designed to evaluate the effectiveness of nirsevimab after its inclusion in the Galicia (Spain) immunization schedule [160]. The study integrated three populations, including infants born during the RSV season, infants younger than 6 months at the start of the RSV season, and infants aged 6–24 months with high‐risk conditions. The results showed that nirsevimab was 82.0% effective against hospitalization for RSV‐associated LRTI. No serious adverse events related to nirsevimab were observed. The NIRSE‐GAL study is currently ongoing with a planned follow‐up period of at least 36 months.

As of February 2025, statistics from 27 observational studies across four countries/regions showed that nirsevimab demonstrated an overall real‐world effectiveness of 75% against RSV associated LRTI, 83% against hospitalizations, and 81% against ICU admissions [161, 162].

#### 3.2.5 MK‐1654 (Clesrovimab)

MK‐1654 is a fully human mAb targeting the F protein IV site (Figure 4A), which derived from the highly potent RB1 antibody against both RSV‐A and RSV‐B strains with a YTE (M252Y/S254T/T256E) mutation in the Fc region to extend half‐life [163]. A phase I placebo‐controlled trial evaluated the safety, tolerability, and pharmacokinetics of MK‐1654 in healthy adults, the results showed that MK‐1654 was well tolerated and safe with a half‐life of 73–88 days [164]. Preliminary data from a phase Ib/IIa study in healthy preterm and term infants, presented in May 2023 at the European Society for Pediatric Infectious Diseases, showed that MK‐1654 had an efficacy of 80.6% in the treatment of RSV LRTI compared with placebo [165, 166]. These clinical results suggest that MK‐1654 holds promise for the treatment of RSV infection in infants. Phase IIb/III trials and phase III trials are currently underway to evaluate the efficacy and safety of MK‐1654 in healthy term and preterm infants.

#### 3.2.6 RSM01

RSM01 is a fully human IgG1 mAb targeting site Ø and also possessed YTE half‐life extended mutations (Figure 4A) [167, 168]. RSM01 was developed for use in low‐ and middle‐income countries (LMICs) [168]. In phase Ia clinical trials, RSM01 was well tolerated and safe in healthy adults [169], and its long half‐life pharmacokinetic profile suggest the potential for dosing once per RSV season in infants [169]. Therefore, RSM01 is expected to provide an effective therapeutic option for infants in LMICs.

### 3.3 Anti‐G mAbs

The G protein, as a key glycoprotein on the surface of RSV, represents an attractive therapeutic target for RSV infection. As a type II transmembrane protein composed of 298–299 amino acids, the G protein features three critical structural domains: a cytoplasmic region, a transmembrane domain (TM), and an extracellular region [23, 170]. The cytoplasmic domain is situated at the N‐terminus of the G protein and contains a crucial cysteine residue that, upon palmitoylation modification, participates in signal transduction [171]. The TM ensures stable anchoring of the protein to the viral envelope. The extracellular region consists of a central conserved domain (CCD), a HS‐binding domain (HBD), and two flanking hypervariable, heavily glycosylated mucin‐like domains. The CCD contains 13 amino acids, which partially overlap with the cysteine noose featuring a 1–4, 2–3 disulfide bond topology. The residues spanning the third and fourth cysteine residues (Cys182–Cys186) form the CX3C motif, which is highly conserved across all strains [172, 173, 174]. This CX3C motif serves as the core functional site of the CCD, capable of mimicking the host chemokine CX3CL1 and binding to the CX3CR1 receptor to mediate viral attachment and immune modulation [172, 173]. Downstream of the cysteine noose lies the HBD, which contains positively charged residues that have been shown to interact with negatively charged HS and other glycosaminoglycans on the host cell surface [175, 176].

During viral infection, the G protein exists in two distinct forms. The membrane‐anchored G protein (mG) possesses 30–40 O‐linked glycans and 3–5 N‐linked glycans, constituting approximately 60% of the G protein's molecular weight [170, 171, 172, 177]. It primarily mediates the initial viral attachment to host cell surfaces, specifically targeting ciliated respiratory epithelial cells and certain immune cells through CX3C–CX3CR1 interactions [178]. Concurrently, the virus secretes substantial amounts of soluble G protein (sG), a truncated variant produced through alternative translation initiation that is released into the surrounding environment early in infection. This sG acts as a “decoy molecule” to neutralize specific antibodies while inhibiting Fc‐mediated antibody‐dependent cellular cytotoxicity (ADCC) and complement activation [178, 179].

As the most variable gene product among all RSV isolates, the G protein's sequence variations have become fundamental to epidemiological and evolutionary studies [23, 180, 181]. The mucin‐like domains form an effective “glycan shield” through dense O‐glycosylation, a unique structural feature that not only masks critical antigenic epitopes but also enables immune evasion through continuously changing glycosylation patterns [181, 182]. This high variability determines RSV's antigenic group classification into two major subtypes: RSV‐A and RSV‐B. Research shows that G proteins from subtypes A and B share only 53% amino acid homology, while sequence variation within the same antigenic group can reach 20% [23, 180, 181]. The recently emerged RSV‐A ON1 variant carries a 72‐nucleotide duplication at the C‐terminus of the G protein, resulting in a 24‐amino acid insertion that may enhance viral adaptability by altering glycosylation patterns or protein conformation. Although some studies associate the ON1 variant with more severe clinical manifestations, the precise mechanisms underlying this relationship require further validation [183, 184, 185].

Despite its high variability, CCD of the G protein offer promising opportunities for therapeutic intervention. Research has shown that mAbs targeting G proteins can not only effectively block viral attachment but also eliminate infected cells through ADCC, playing a crucial role in antiviral immunity [172, 186, 187, 188].

#### 3.3.1 131–2G

The central conserved region of the RSV G protein contains a CX3C chemokine motif, which is associated with modulating the virus‐induced host immune response [188]. The mAb 131–2G specifically targets the CX3C motif, preventing RSV infection by blocking the binding of the G protein to the CX3CR1 receptor (Figure 4B) [189]. The intact component of 131–2G reduces viral replication, while its F(ab′)2 component specifically attenuates virus‐induced pulmonary inflammation [190]. In RSV‐infected BALB/c mice, 131–2G treatment ameliorated multiple disease manifestations, including pulmonary inflammation, BAL leukocyte infiltration, pulmonary mucin levels, airway reactivity, Th2‐type cytokine production, weight loss, and breathing effort [189, 191]. Prophylactic administration of 131–2G also attenuated the formalin inactivated RSV (FI‐RSV) enhanced pulmonary inflammation in mice [192]. 131–2G has significant advantages in treating disease during RSV infection, which may be more effective than just blocking viral replication, as suggested by anti‐F‐protein mAbs like palivizumab [193].

#### 3.3.2 3D3 and 2B11

3D3 and 2B11 are mAbs derived from B cells of RSV‐infected adults that bind the same antigenic region as 131–2G (Figure 4B) [194]. Several studies have shown that 3D3 and 2B11 are more effective than anti‐F‐protein mAbs in reducing LRTI, pulmonary inflammation, and proinflammatory factor production during RSV infection [187, 194, 195]. In addition, 3D3 was shown to neutralize viruses in primary cell culture systems, which may contribute to the anti‐inflammatory properties of anti‐G mAbs [194].

### 3.4 Nanobody

Nanobodies are therapeutic proteins derived from the variable domains of heavy‐chain antibodies found in camelids, with a molecular weight of approximately 15 kDa [196]. They offer unique advantages, including a simple structure, high stability, and superior tissue penetration. Due to their small size and extended CDR3 loops, nanobodies can bind to conformational epitopes that may be inaccessible to conventional antibodies and can be flexibly engineered into multivalent or multispecific molecules. Their exceptional stability allows for administration via nebulization without loss of activity, demonstrating certain prospects in the treatment of infectious diseases [197, 198, 199].

ALX‐0171 (Gontivimab) is a trimeric nanobody that binds antigenic site II of the F protein (Figure 4A), partially overlapping with the epitope recognized by palivizumab [198]. ALX‐0171, only 42 kDa in size, can be inhaled by nebulization directly into the lungs [198]. Preclinical studies demonstrated that formatting the monovalent building block of ALX‐0171 into a trivalent format significantly increased its ability to neutralize RSV‐A and RSV‐B strains by over 6000‐fold [197]. Compared with palivizumab, ALX‐0171 was 126‐fold and sixfold more potent against RSV‐A Long and RSV‐B 18537, respectively [197]. In vivo, ALX‐0171 effectively reduced or blocked RSV replication in the lungs and nose of cotton rats [197]. In a neonatal lamb model, ALX‐0171‐treated lambs showed a significant reduction in RSV viral titers, pulmonary lesions, histopathology, and clinical severity [200]. Notably, optimized nebulization is a significant advantage of ALX‐0171, as targeted efficacious concentrations of ALX‐0171 in pulmonary epithelial lining fluid can be readily achieved by nebulized administration [200]. Moreover, ALX‐0171 showed considerable neutralizing potency against most escape mutants in escape mutants studies, making it an attractive option for considering viral mutations driven by therapeutic interventions [199]. In a multicenter phase I/IIa study, nebulized ALX‐0171 administered to 53 infants hospitalized for RSV LRTI resulted in a significant reduction in intranasal viral titers with no safety concerns [201]. However, a phase IIb clinical trial revealed that although ALX‐0171 achieved rapid reduction in nasal viral load, no significant difference in primary clinical endpoints was found when compared with placebo [201]. Due to insufficient clinical efficacy, ALX‐0171 development was discontinued. The fundamental limitations of ALX‐0171 as a nanobody stem from its lack of an Fc domain, which precludes effector functions like ADCC, and its inability to neutralize intracellular viruses [202]. Moreover, compared with contemporaneous intramuscularly administered nirsevimab, ALX‐0171 required nebulized inhalation in infants, resulting in poor clinical compliance and operational inconvenience [202]. Consequently, future nanobody development should incorporate Fc fusion or other protein engineering strategies to restore immune effector functions while simultaneously optimizing delivery systems for enhanced clinical utility (Tables 1 and 2).

**TABLE 1:** Clinical development status of antibodies targeting RSV.

Antiviral class	Drug candidate	Company	Virus target	Development phase	Target population
Immunoglobulin	Respigam (RSV‐IVIG)	MedImmune	High‐titer RSV neutralizing antibodies	Phase IV, discontinued	High‐risk infants [120]
	RI‐001	ADMA biologics		Phase II, completed	Immunocompromised [127]
	RI‐002	ADMA biologics		Approved in 2019	Patients with PIDD [128]
Monoclonal antibodies	Palivizumab (Synagis)	MedImmune	F protein, Site II	Approved in 1998	High‐risk infants [141]
	Motavizumab (MEDI‐524)	MedImmune	F protein, Site II	Phase III, not approved by US FDA due to security concerns	High‐risk infants [144]
	Suptavumab (REGN2222)	Regeneron	F protein, Site V	Phase III, failed due to lack of efficacy	Preterm infants [151]
	Nirsevimab (Beyfortus)	AstraZeneca & Sanofi	F protein, Site Ø	Approved in 2022	Neonates and infants [159]
	MK‐1654 (Clesrovimab)	Merck	F protein, Site IV	Phase III, ongoing	Healthy preterm and full‐term infants [165]
	RSM01	Gates MRI	F protein, Site Ø	Phase Ia, completed	Infants in low‐ and middle‐income countries [168]
	131‐2G [189]	MedImmune	G protein	/	/
	3D3 and 2B11 [194]	/	G protein	/	/
Nanobodies	ALX‐0171 (Gontivimab)	Ablynx	F protein, Site II	Phase IIb, failed due to lack of efficacy	Infants [201]

Abbreviation: PIDD, primary immunodeficiency disease.

**TABLE 2:** Clinical development status of small‐molecule antiviral candidates targeting RSV.

Drug candidate	Company	Virus target	Development phase	Study population
Presatovir (GS‐5806)	Gilead	F protein	Phase II, failed due to lack of efficacy	Healthy adults, HSCT recipients (URTI and LRTI), lung transplant [220, 221, 222, 223]
JNJ‐53718678 (Rilematovir)	Johnson & Johnson	F protein	Phase II, discontinued	Healthy adults, infants [224, 225, 226, 227]
JNJ‐2408068	Johnson & Johnson	F protein	Preclinical [228]	/
TMC353121	Janssen	F protein	Preclinical [232]	/
BTA‐C585 (Enzaplatovir)	Aviragen Therapeutics	F protein	Phase IIa, discontinued due to lack of efficacy	Healthy adults [235]
MDT‐637 (VP‐14637)	Gilead	F protein	Phase IIa, discontinued due to lack of efficacy	Healthy adults [235]
AK‐0529 (Ziresovi, RO‐0529)	Ark Biosciences	F protein	Phase III, completed; NDA in China	Infants [242, 243, 244]
RV521 (Sisunatovir)	ReViral	F protein	Phase II, discontinued	Healthy adults, infants, elderly, immunocompromised [247]
Lonafarnib	Medizinische Hochschule Hannover	F protein	Preclinical [250]	/
ALS‐8176 (Lumicitabine)	Alios BioPharma	Polymerase	Phase IIb, discontinued due to security concerns	Healthy adults, neonates and infants [257, 259, 260]
Remdesivir (GS‐5734)	Gilead Sciences	Polymerase	Preclinical [262]	/
PC786	Pulmocide	Polymerase	Phase II, discontinued	Healthy adults, HSCT recipients [267]
AZ‐27	Astra Zeneca	Polymerase	Preclinical [268]	/
JNJ‐8003	Johnson& Johnson	Polymerase	Preclinical [270]	/
BI‐D	Boehringer Ingelheim	Polymerase	Preclinical [273]	/
DZ7487	Dizal Pharmaceuticals	Polymerase	Preclinical [275]	/
RSV604	Astra Zeneca	N protein	Phase IIa, discontinued due to lack of efficacy	HSCT recipients [280]
ALN‐RSV01	Alnylam	N protein	Phase IIa and IIb, discontinued due to lack of efficacy	Healthy adults, LTx recipients [283, 284, 287, 288]
EDP‐938 (Zelicapavir)	Enanta	N protein	Phase II, ongoing	Healthy adults, infants, HSCT recipients [291]
Verdinexor (KPT‐335)	Karyopharm	M protein	Phase I, completed	Healthy adults [306]
CPM and A3E	Université Paris‐Saclay	M2‐1 protein	Preclinical [317]	/
AT‐2	Virion Systems, Cardiff University	M2‐1 protein	Preclinical [310]	/

Abbreviations: HSCT, hematopoietic stem cell transplant; LRTI, lower respiratory tract infection; LTx, lung transplant; URTI, upper respiratory tract infection.

## 4 Small Molecular Antivirals Against RSV

Current development of RSV‐specific small molecule antivirals mainly focuses on two principal approaches: (i) Direct‐acting antivirals targeting key viral lifecycle proteins, including small‐molecule inhibitors that block viral entry, transcription, replication, assembly, and release; and (ii) host‐targeting antivirals that function by modulating host immune responses, inflammatory pathways, and cellular defense mechanisms. Notably, several innovative drug candidates have entered global clinical development pipelines, demonstrating transformative therapeutic potential.

### 4.1 Targeting F Protein

The F protein, a class I viral fusion glycoprotein, is essential for viral entry into host cells. The F protein exhibits high conservation between different RSV subtypes, with only approximately 5% sequence variation between subtypes A and B [76], making it one of the most promising targets for current anti‐RSV drug development. Initially synthesized in the endoplasmic reticulum as a 574‐amino‐acid precursor (F0), the F protein is cleaved by furin protease in the Golgi apparatus, removing a central 27‐amino‐acid fragment (p27) to generate F1 and F2 subunits [203, 204, 205]. The F2 subunit, located at the N‐terminus, contains the heptad repeat C (HRC) region. The F1 subunit at the C‐terminus features a central trimeric hydrophobic pocket as its core structural element. This pocket, approximately 10 Å in diameter and 15 Å deep, encapsulates the fusion peptide (FP), while three HRA domains surround the central cavity, and the heptad repeat B (HRB) forms a stable triple‐helical bundle at the base, collectively maintaining the metastable prefusion conformation. Additionally, the F1 subunit contains cysteine‐rich regions (Domains I/II), a TM, and a cytoplasmic tail [23, 138, 203, 204, 206, 207]. The F2 and F1 subunits are covalently linked by two disulfide bonds to form a heterodimeric protomer, with three protomers noncovalently assembling into a trimer (mature F protein) [205, 208].

Unlike most viral fusion proteins, RSV F protein activation is pH‐independent (functional within pH 5.5–8.5) and does not require assistance from other viral glycoproteins [70, 209, 210]. Fusion initiation may occur through cholesterol‐rich membrane microdomains or macropinocytosis [23, 211]. During fusion, the FP is released from the central hydrophobic cavity and inserts into the target cell membrane, while the HRA domains extend from compact loop structures into approximately 70 Å‐long α‐helices. Concurrently, the HRB domains dissociate from the parallel helical bundle at the trimer base and ultimately wrap around HRA in an antiparallel manner to form an exceptionally stable six‐helix bundle (6‐HB) structure [207, 212, 213]. This irreversible transition from the pre‐F to post‐F state not only brings viral and cellular membranes into close proximity but also provides the free energy required to drive fusion pore formation, creating essential conditions for viral genome release [204, 212, 213, 214].

In contrast to mAbs that target specific epitopes on the F protein surface, most F protein inhibitors (e.g., presatovir and JNJ‐53718678) bind to a triple‐symmetric pocket in the central cavity of the pre‐F conformation (Figure 3C). This pocket is formed by highly conserved amino acid residues with distinct hydrophobic and geometric characteristics [139]. Multiple small‐molecule inhibitors targeting the F protein have been successfully developed, employing distinct mechanisms such as stabilizing the pre‐F conformation, blocking HRA–HRB interaction, and disrupting conformational changes [215, 216, 217].

#### 4.1.1 Presatovir (GS‐5806)

Presatovir is an oral fusion inhibitor that targets the triple‐symmetric pocket in the central cavity of the pre‐F conformation, blocking the RSV conformational change from pre‐F to post‐F [215, 218]. In an in vitro study, presatovir was tested on 75 clinical isolates of RSV‐A and RSV‐B, demonstrating reduced viral replication in all isolates tested (EC_50_, 0.1–1.2 nM) and dose‐dependent antiviral efficacy [219]. In a phase IIa study in healthy adults, presatovir treatment reduced viral load, total mucus production, and total symptom scores [220]. Subsequently, three phase IIb studies assessed the efficacy of presatovir in immunocompromised populations, including hematopoietic cell transplant recipients with URTI/LRTI, and LTRs, the results showed that presatovir did not significantly improve patients’ nasal RSV loads or clinical outcomes [221, 222, 223]. Although clinical endpoints were not met in these three immunocompromised populations, further clinical trials of presatovir in different populations may be warranted.

#### 4.1.2 JNJ‐53718678 (Rilematovir)

JNJ‐53718678 is another highly active RSV fusion inhibitor against a wide range of RSV subtypes A and B strains, with a mean EC_50_ of 0.46 nM, which blocks viral invasion of host cells by binding to pre‐F like presatovir [216]. In rodents and neonatal lambs, JNJ‐53718678 effectively inhibited RSV transmission and RSV‐induced lung inflammation and disease [216]. A phase IIa human challenge study evaluated the antiviral activity of different doses of JNJ‐53718678 (75, 200, and 500 mg) [224]. Lower viral loads, mean overall symptom scores, and nasal secretion weights were observed in the JNJ‐53718678‐treated group at each dose compared with the placebo group, and no clear dose–response relationship was observed [224]. In a phase Ib study in RSV‐infected hospitalized infants [225] and a phase IIa study in RSV‐infected nonhospitalized adults [226], JNJ‐53718678 treatment was safe and well tolerated, exhibiting clinical benefit in terms of antiviral activity. Another phase II CROCuS study evaluating the efficacy and safety of rilematovir in children with RSV infection was terminated before completion [227]. Compared with the placebo, rilematovir exhibited a small but favorable antiviral effect and demonstrated good safety. Due to a nonsafety‐related strategic decision made by the sponsor, the development of rilematovir has been discontinued [227].

#### 4.1.3 JNJ‐2408068

JNJ‐2408068 is a low molecular weight benzimidazole derivative with a dual mode of action: inhibition of virus–cell fusion early in the infection cycle and cell–cell fusion at the end of the replication cycle [228]. JNJ‐2408068 specifically binds to the hydrophobic groove of the HRA and HRB domain, thereby disrupting the formation of the HRA–HRB 6‐HB [217]. The antiviral activity of JNJ‐2408068 has been confirmed using human RSV‐A and RSV‐B clinical isolates, as well as in bovine RSV isolates [228]. The EC_50_ of JNJ‐2408068 (0.16 nM) is 100,000 times more potent than ribavirin (15 µM) against RSV‐A Long strain [228]. JNJ‐2408068 also inhibited the release of proinflammatory cytokines IL‐6, IL‐8, and secretion from RSV‐infected A549 cells [228]. A preclinical study showed that JNJ‐2408068 protected cotton rats from experimental RSV infection without exhibiting toxic effects [229].

#### 4.1.4 TMC353121

TMC353121, a derivative of JNJ‐2408068, was developed through structural modifications to address the lacked long tissue retention of its predecessor [230]. TMC353121 binds to HRA and HRB domains, thereby inhibiting the assembly of the fusion‐required 6‐HB [231]. In BALB/c mice, TMC353121 (0.25–10 mg/kg) significantly reduced viral load, bronchoalveolar lavage cell accumulation, and the severity of histopathologic changes in the lungs [232]. Furthermore, TMC353121 displayed dose‐dependent antiviral activity in nonhuman primates, and complete inhibition of viral shedding was observed at lower plasma exposures (0.39 µg/mL), which was accompanied by decreased level of inflammatory cytokines, including INFγ, IL6, and the chemokine MIP1α [233].

#### 4.1.5 BTA‐C585 (Enzaplatovir)

BTA‐C585 inhibits the replication of RSV‐A and RSV‐B clinical isolates in vitro with an EC_50_ of about 100 nM [234]. BTA‐C585 has shown antiviral activity against RSV in the cotton rat model [234], highlighting its promising therapeutic potential. A randomized, placebo‐controlled phase IIa clinical trial evaluated the safety and efficacy of BTA‐C585 in healthy adult volunteers, and the results showed no significant difference in efficacy between the placebo group and the treatment group [235]. The clinical development of BTA‐C585 was terminated after the phase II trial.

#### 4.1.6 MDT‐637 (VP‐14637)

MDT‐637 is a derivative of VP‐14637, which exhibits nanogram‐range inhibition of clinical strains encompassing diverse RSV genotypes and clades [236]. VP‐14637 shares a similar anti‐RSV mechanism with JNJ‐2408068, targeting a small hydrophobic cavity in the core of the F protein while interacting with both HRA and HRB domains [217, 236]. Moreover, significant binding of MDT‐637 to the F protein occurs exclusively at 37°C, while its binding is markedly impaired at 22°C or below. This temperature‐dependent binding profile suggests that VP‐14637 likely recognizes transient intermediate states during the conformational transition of the F protein [236]. MDT‐637 is administered as a pulmonary dry powder inhaler, enabling rapid drug delivery to the respiratory tract [237]. In vitro studies reveal that MDT‐637 possesses antiviral potency ranging from hundreds to thousands of times greater than ribavirin, suggesting its potential for superior clinical outcomes in the treatment of natural RSV infections [238]. A phase 2a trial initiated in 2013, was conducted to evaluate the antiviral effects of nebulized MDT‐637 among healthy participants, while it was subsequently halted due to poor efficacy [235]. The clinical development of MDT‐637 has been discontinued.

#### 4.1.7 AK‐0529 (Ziresovir, RO‐0529)

AK‐0529 inhibits viral fusion by specifically targeting the D486 and D489 residues (resistance mutation sites: D486N, D489A/V/Y) in the C‐terminal HRC region of the RSV F protein [239]. AK‐0529 exhibits RSV‐specific antiviral activity, with EC_50_ values in single‐digit nM levels, as demonstrated by testing against multiple RSV‐A and RSV‐B strains [239]. Oral administration of AK‐0529 showed a strong antiviral effect (>1 log unit reduction in viral titers in lungs) in the BALB/c mouse model of RSV infection [239]. A placebo‐controlled phase I study showed that oral administration of AK‐0529 was well tolerated and safe [240]. In a phase II (VICTOR) study involving 1–24 months infants hospitalized for RSV infection, AK‐0529 treatment improved clinical signs and symptoms RSV induced capillary bronchiolitis leading to a reduced viral load, and no safety or tolerability issues were observed for AK‐0529, and there were no deaths or withdrawals from the study [241]. These favorable outcomes lend credence to the conduct of phase III clinical trial (AIRFLO), and Ark Biosciences announced the results of study in December 2022 [242, 243, 244]. The study evaluated the efficacy and safety of AK‐0529 in the treatment of hospitalized infants aged 1–24 months with RSV infection. Compared with the placebo group, AK‐0529 improved clinical symptom scores for capillary bronchiolitis by 30% on day 3 and reduced viral load by 77% on day 5, with no safety concerns were identified. Furthermore, In RSV‐infected ICU patients, AK‐0529 significantly shortened ICU treatment duration, but its impact on the ICU admission rate remained unclear. In addition, follow‐up data collected 6 months posttreatment revealed a significantly low proportion of recurrent wheezing in children aged 6 months and younger treated with AK‐0529 compared with the placebo group (9.0 vs. 26.3%). During the 24‐month follow‐up, treatment with ziresovir reduced wheezing‐related hospital visits, the frequency of wheezing episodes, and the incidence of asthma in children aged 6 months or younger. The observed adverse events were mostly mild reactions such as diarrhea and rash, with no drug‐related serious adverse events or deaths reported [245]. Based on positive phase III results, China's National Medical Products Administration approved Priority Review of New Drug Application (NDA) for AK‐0529 to treat RSV infection in December 2022. AK‐0529 will be further explored in other populations, such as preterm infants, high‐risk infants with CHD or BPD, and so on.

#### 4.1.8 RV521 (Sisunatovir)

RV521 is an orally available small molecule fusion inhibitor, identified through a lead optimization process based upon hits from property directed hit profiling exercise at Reviral [246]. RV521 specifically targets the central binding pocket of the RSV F protein prefusion trimer, with critical interactions at Phe140/Phe488 (π–π stacking) and Thr397 (hydrogen bonding) [246]. RV521 demonstrated potent antiviral activity against various RSV‐A and RSV‐B laboratory strains and clinical isolates, with a mean EC_50_ value of 1.2 nM. In the BALB/c mouse model, virus titers in the lungs of 10 and 50 mg/kg RV521‐treated mice were reduced by more than 1 log10 compared with the control group [246]. In a phase IIa study in healthy adults, both 350 and 200 mg doses of RV521 effectively reduced RSV viral load and disease severity, and were well tolerated and safe [247]. The two phase II clinical trials of RV521 had received US FDA Fast Track designation for the treatment of RSV‐associated LRTI in infants and RSV‐associated URTI in adult hematopoietic cell transplant recipients, respectively. However, both trials were terminated due to strategic considerations and other unknown reasons [248].

#### 4.1.9 Lonafarnib

Lonafarnib, an approved farnesyltransferase inhibitor for Hutchinson–Gilford progeria syndrome, was identified as an RSV F protein inhibitor via a drug repurposing strategy [249, 250]. Lonafarnib binds to the triple‐symmetric pocket within the central cavity of RSV F protein's metastable prefusion conformation and interacts with the FP, thereby preventing its insertion into the host cell membrane [250]. Lonafarnib treatment reduced syncytium formation in infected and F protein overexpressing cells, with an EC_50_ ranging from 10 to 118 nM. Furthermore, lonafarnib reduced RSV viral load in BALB/c mice in a dose‐dependent manner [249, 250]. However, lonafarnib demonstrates comparatively lower efficacy than clinical‐stage RSV inhibitors, and its farnesyltransferase inhibitory activity may induce off‐target effects, particularly at higher oral doses [250].

### 4.2 Targeting RSV Polymerase

The RSV polymerase is composed of the L protein and the P protein, which plays a crucial role in RNA synthesis. The RSV polymerase recognizes the promoter at the 3′‐terminus of both the viral genomic RNA and antigenomic RNA, corresponding to the leader and trailer complement sequences, respectively. A distinctive feature of the RSV polymerase is its ability to initiate RNA synthesis from two distinct positions within these promoters (position +1 or position +3), with these alternative initiation sites corresponding to genome replication and mRNA transcription, respectively [251].

The L protein is a multifunctional RdRp consisting of five domains: the RdRp domain, the capping domain (Cap), the connector domain (CD), the methyltransferase domain (MT), and the carboxy‐terminal domain (CTD). Among these, the RdRp, Cap, and MT domains are the core structural domains essential for viral RNA synthesis [251]. The RdRp domain adopts a right‐handed structure, with three subdomains—palm, thumb, and fingers—forming a catalytic center. It contains three conserved regions (CR_I, CR_II, and CR_III) and seven conserved motifs (A–G), which mediate the initiation and elongation of RNA strand synthesis [252, 253]. Notably, motif B harbors a conserved Asn residue that assists in substrate positioning and template translocation. Through allosteric effects, motif B can alter the conformation of the polymerase active site, enabling the virus to discriminate between natural nucleotides and nucleotide analogs, making it a potential drug target [254, 255]. The Cap domain participates in the 5′ mRNA capping process, where conformational changes in its priming loop (residues 1267–1282) are closely associated with transcriptional state transitions. The MT domain catalyzes the methylation of the cap structure [254].

The P protein acts as a cofactor for the L protein, anchoring it to the nucleoprotein–RNA complex and regulating viral RNA replication and transcription. The P protein is divided into three domains: the N‐terminal domain, the oligomerization domain, and the C‐terminal domain [251, 254]. Cryo‐EM studies have revealed the structure of the L–P complex, showing that the P protein exists as a unique tetramer, with its central oligomerization domain forming a stable coiled‐coil core. The four monomers of the P protein adopt distinct spatial arrangements, wrapping around the L protein in an asymmetric “tentacle‐like” conformation. This multiconformational flexibility allows the P protein to interact with multiple regions of the L protein. Additionally, the RdRp and Cap domains of the L protein exhibit an intertwined spatial relationship, suggesting that the initiation of RNA synthesis by the polymerase may be regulated by the Cap domain [254]. The high‐resolution structure of the RSV polymerase complex provides a critical foundation for understanding viral replication mechanisms and developing targeted antiviral drugs.

#### 4.2.1 ALS‐8176 (Lumicitabine)

ALS‐8176 is a nucleoside analog prodrug that exerts its effects on the L–P complex of RdRp [256]. In plasma, this prodrug undergoes metabolic transformation to ALS‐8112, which then undergoes conversion in host cells to the 5'‐triphosphate metabolite (ALS‐8112‐TP), an active inhibitor of RSV polymerase [257]. Biochemical studies have shown that the recombinant RSV polymerase complex efficiently recognizes ALS‐8112‐TP, leading to chain termination of RNA synthesis. RSV develops resistance to ALS‐8176 through a quadruple mutation cluster (M628L/A789V/L795I/I796V) in the L protein, with these substitutions located within the α29 helix of the polymerase Motif B [254, 256]. ALS‐8176 effectively inhibits RSV replication in nonhuman primates in vivo and against all RSV strains in vitro [258]. In addition, ALS‐8176 exhibits broad‐spectrum inhibition against paramyxovirus, particularly with specificity toward paramyxoviruses and elastoviruses [258].

Two clinical studies with healthy volunteers infected with RSV show that intranasally inoculated ALS‐8176 extensively and rapidly suppressed RSV replication and reduced viral load with good tolerance [257, 259]. Specially, the highest antiviral activity was found in the two groups that received the loading dose (LD) of 750 mg. Moreover, the secondary endpoints, including time to undetectable virus (1.3–2.3 days), clearance of RSV RNA (slope of viral load), peak viral load, RSV‐related symptom scores, and the AUC for mucus weight, were also significantly lower in the treated groups than those in the placebo group, with no serious adverse events observed. Subsequent phase Ib and phase IIb studies were conducted to assess the safety and efficacy of ALS‐8176 in RSV‐infected hospitalized infants or neonates [260]. In the phase Ib study, infants (1–12 months) and neonates (<28 days) were randomized in a 3:1 ratio to receive either a single ascending dose or multiple ascending doses (single LD followed by 9 maintenance doses [MD]) of ALS‐8176 or placebo. In the phase IIb study, infants and children (28 days–36 months) were randomized in a 1:1:1 ratio to receive ALS‐8176 40/20 mg/kg LD/MD, 60/40 mg/kg LD/MD twice daily, or placebo for 5 days. In both studies, there were no significant differences between the ALS‐8176‐treated and placebo groups in terms of reduction of nasal RSV viral load, time to viral clearance, or RSV symptom remission, which is contrary to the results of two previous RSV‐A healthy adult challenge studies [257, 259]. In addition, reversible neutropenia was observed during treatment, its incidence and severity was associated with a dose‐dependent increase in ALS‐8176 [260]. The sponsor suspended the phase IIb human clinical study. Subsequent studies in nonhuman primates revealed potential hematopoietic and clastogenic effects at lower blood concentrations of ALS‐8176 [260]. Due to this adverse effect and the lack of efficacy in RSV‐infected hospitalized infants, Alios BioPharma decided to terminate the development of ALS‐8176.

#### 4.2.2 Remdesivir (GS‐5734)

Remdesivir is a broad‐spectrum antiviral nucleoside analog that exerts its antiviral effects by inhibiting the RSV RdRp [261]. Mechanistically, remdesivir in its active triphosphate form (RDV‐TP) competitively incorporates into the RSV RdRp, subsequently inducing delayed chain termination five nucleotides downstream (i+5 position), thereby effectively blocking viral RNA synthesis [261]. In vivo, remdesivir reduced RSV viral load in cynomolgus and African Green monkeys [262]. In addition, remdesivir has been shown to be effective against the RNA polymerases of EBOV, SARS‐CoV, and ERS‐CoV [263]. Given the high sequence and structural homology between RSV RdRp and the catalytic subdomain of the EBOV RNA polymerase [237], remdesivir may also be therapeutically effective against RSV.

#### 4.2.3 PC786

PC786 is a non‐nucleoside inhibitor targeting the RSV L protein polymerase, exhibiting potent antiviral activity. PC786 demonstrates sub‐nanomolar efficacy against RSV‐A (EC_50_, 0.09–0.71 nM) and RSV‐B (EC_50_, 1.3–50.6 nM) [264]. Recent studies revealed that PC786 not only induces the Y1631H mutation in the L protein but also triggers the V153A mutation in the M protein, leading to the emergence of escape mutants [265]. By targeting the 1392–1735 amino acid region of the L protein, PC786 disrupts L–M protein interaction, resulting in increased nuclear localization of M protein and delayed positioning of the F protein at viral budding filaments. These findings suggest that the L–M protein interaction may represent a novel therapeutic target for RSV antiviral development [265]. Unlike the F‐protein inhibitor GS‐5806, the antiviral efficacy of PC786 was virtually unaffected by the multiplicity of infection [264]. Topical administration of PC786 to the lungs of RSV‐infected mice or cotton rats significantly reduced the viral load [264]. Compared with the RSV nucleoside analog ALS‐8112 and the F‐protein inhibitor GS‐5806, late therapeutic intervention with PC786 rapidly reduced viral load in human airway epithelial cells, demonstrating superior antiviral properties [266]. In healthy subjects infected with RSV‐A (Memphis 37b), nebulized PC786 was well tolerated and exhibited significant antiviral effects with a trend toward lower symptom scores and mucus weights [267]. However, A phase II double‐blind, placebo‐controlled trial in adult HSCT recipients was interrupted for unknown reasons.

#### 4.2.4 AZ‐27

AZ‐27 is designed based on YM‐53403, identified through CEP screening efforts. AZ‐27 exhibited higher potency against RSV‐A (EC_50_, 6–14 nM) and RSV‐B (EC_50_, 0.4–2.2 µM) strains compared with YM‐53403 [268]. AZ‐27 has been shown to affect RSV polymerase activity by inhibiting mRNA transcription and genome replication, especially targeting the initiation of RNA synthesis at the promoter [269]. Currently, there are no in vivo or clinical trials related to AZ‐27.

#### 4.2.5 JNJ‐8003

JNJ‐8003 is a non‐nucleoside inhibitor targeting the RSV polymerase, demonstrating potent subnanomolar inhibitory activity (EC_50_<1 nM) [270, 271]. JNJ‐8003 inhibits nucleotide polymerization during the early stages of RNA transcription and replication by modulating the functional crosstalk between the Cap domain and RdRp domain. Specifically, JNJ‐8003 binds to an induced‐fit pocket in the Cap domain, forming multiple interactions with key residues including L1337 and H1338, which completely shuts down the RdRp function [270, 271]. JNJ‐8003 has exhibited significant antiviral efficacy in both mouse and neonatal lamb models, showing promising clinical development potential [272].

#### 4.2.6 BI‐D

BI‐D is another non‐nucleoside inhibitor targeting the Cap domain of the RSV polymerase. Resistance mutation analysis indicates that BI‐D has three resistance‐associated amino acid substitutions (I1381, L1421, and E1269) in the Cap domain [254, 273]. Among them, I1381 and L1421 are located at adjacent positions on the surface of the Cap domain, together forming a small surface pocket (comprising motifs B, D, and E), while E1269 indirectly affects the BI‐D binding pocket through an allosteric effect, thereby conferring resistance. BI‐D not only inhibits mRNA 5′ capping but also abnormally enhances polymerase elongation activity, thereby interfering with normal transcription and replication processes [274]. In RSV‐infected BALB/c mice, intranasal administration of BI‐D resulted in a significant reduction in RSV viral load in the lungs [273].

#### 4.2.7 DZ7487

DZ7487 is a novel oral non‐nucleoside inhibitor targeting the highly conserved RdRp domain of the RSV polymerase. Resistance profiling revealed that the primary resistance mutation for DZ7487 is located at the N363T position within the RdRp domain. DZ7487 demonstrates inhibitory activity against multiple clinical isolates of both RSV A and B subtypes, exhibiting potent antiviral efficacy both in vitro and in vivo. DZ7487 demonstrates significant viral load reduction even after symptom onset, showing promise as an oral therapeutic for RSV infection [275].

### 4.3 Targeting N Protein

The N protein encapsidates viral RNA to form RNPs, thereby effectively protects the viral genome from degradation. Comprising 391 amino acids, the N protein consists of two core domains: the N‐terminal domain (NTD) and C‐terminal domain (CTD), along with the N‐terminal motif (N‐arm) and C‐terminal motif (C‐arm) near the ends of the core domains [61, 276]. A central RNA‐binding groove, located between the NTD and CTD, interacts with the viral genomic RNA through positively charged amino acid residues, forming a left‐handed helical nucleocapsid [84, 276]. The NTD of the RSV N protein interacts with the P protein, promoting RNP stability and recruiting the viral polymerase complex [61, 277]. Due to its high conservation across all known RSV subtypes, the N protein represents an ideal target for the development of broad‐spectrum anti‐RSV therapeutics.

#### 4.3.1 RSV604

RSV604 is an oral 1,4‐benzodiazepine with submicromolar anti‐RSV activity which is effective when administered postinfection [278]. In the HAE cell model, RSV604 administered 24 h after infection effectively inhibited viral replication and transmission [278]. In HeLa cells, RSV604 inhibited RSV RNA synthesis and the infectivity of the released virus. However, RSV604 had no significant effect on viral RNA synthesis in RSV replicon cells and cell‐free RNP assays, possibly because it inhibits the initiation of the assembly of functional viral replication complexes rather than acting on replication complexes already formed within cell [279]. In a phase IIa trial involving stem cell transplant recipients infected with RSV, reductions in viral load and symptoms were observed only in patients with RSV604 plasma levels up to EC_90_ [280]. Most patients did not exhibited significantly different viral loads compared with the placebo group, partly due to the small sample size, the complexity of the underlying conditions, and the seasonality of the virus [280]. Development of RSV604 has been discontinued due to poor efficacy.

#### 4.3.2 ALN‐RSV01

Small interfering RNAs (siRNAs) degrade mRNAs in a sequence‐specific manner via RNA interference, thereby reducing the expression of the corresponding proteins [281]. ALN‐RSV01 is a double‐stranded siRNA targeting the mRNA of the RSV N protein with 19 paired nucleotides, which demonstrated specific antiviral activity both in vitro and in vivo [282]. In phase I trials, the intranasal administration of ALN‐RSV01 was shown to be safe and well tolerated [283]. In a phase II trial in healthy adults, ALN‐RSV01 significantly reduced viral activity in subjects, resulting in a 38% reduction in infected rates and a 95% increase in uninfected individuals in the ALN‐RSV01 group compared with the placebo group [284]. RSV‐induced LRTI has been strongly associated with the development of bronchiolitis obliterans syndrome (BOS) in LTx recipients [285, 286]. To evaluate the efficacy and safety of ALN‐RSV01 in LTx recipients infected with RSV, phase IIa and IIb clinical studies were conducted [287, 288]. The incidence of new or progressive BOS was reduced in the ALN‐RSV01‐treated group compared with placebo, while no statistical significance was found in viral parameters and symptom scores. Currently, the development of ALN‐RSV01 is not being further pursued.

#### 4.3.3 EDP‐938 (Zelicapavir)

EDP‐938 was discovered through a series of chemical optimizations, stemming from the 1,4‐benzodiazepine RSV inhibitors which exhibited high antiviral activity (EC_50_, 21–64 nM) against all RSV laboratory strains and clinical isolates [289]. In vitro resistance studies have shown that EDP‐938 possesses a higher resistance barrier compared with fusion inhibitors and RSV polymerase inhibitors. EDP‐938 maintains cytoprotection and RNA inhibition even after RSV infection, indicating its efficacy at the replication step after viral entry [289]. A study evaluated the development of resistance to EDP‐938 in human trials: only 5% of treated participants developed resistance mutations, and these mutant strains were rapidly cleared due to reduced viral fitness, whereas the comparator fusion inhibitor presatovir showed a much higher resistance rate of 28% [290]. This high resistance barrier highlights the significant advantage of N‐protein‐targeting inhibitors. In a phase IIa clinical study involving healthy adults, EDP‐938 outperformed placebo in terms of viral load, total symptom score, and mucus weight. Furthermore, there were no significant safety were observed across various dosing regimens of EDP‐938 [291]. A phase II study in hospitalized and nonhospitalized children, and a phase IIb study in adult HSCT recipients with acute RSV infection and URTI are currently underway.

### 4.4 Targeting M Protein

The M protein is a nonglycosylated, phosphorylated protein composed of 256 amino acids and serves as a critical structural component of the viral particle [292]. The hydrophobic profile of RSV M protein resembles that of other mononegavirus M proteins: the N‐terminal region exhibits low hydrophobicity, while the C‐terminal region displays significantly enhanced hydrophobicity. Although the hydrophobic regions are too short to form TMs, they likely facilitate peripheral attachment to the host cell membrane [23, 87, 292]. Key functional domains include the central nucleic acid‐binding domain (containing a nuclear localization signal) and the C‐terminal nuclear export signal [23, 292]. Crystallographic analysis reveals that the M protein exists as a dimer and further assembles into higher‐order oligomers. This dynamic oligomerization of M protein serves as the key driving force for viral budding [293].

During viral assembly and budding, M protein plays a pivotal role by bridging viral RNPs and envelope glycoproteins, thereby coordinating virion formation [294]. It also interacts with host lipid rafts and cytoskeletal proteins (e.g., actin, cofilin‐1, caveolin‐1, and Rab11a) to promote viral budding [295, 296, 297, 298]. Notably, M protein exhibits specific membrane targeting—while it can bind to the plasma membrane when expressed alone, efficient localization to lipid raft microdomains requires coexpression with the F protein [87]. Furthermore, M protein shuttles between the nucleus and cytoplasm: during early infection, it enters the nucleus via importin β1‐dependent nuclear import, while later in infection, it returns to the cytoplasm through CRM1 (i.e., Chromosome Region Maintenance 1; also known as exportin 1 or XPO1)‐mediated nuclear export to participate in viral assembly [299, 300, 301]. Although the precise mechanistic role of M protein in the nucleus remains to be fully elucidated, its nuclear accumulation has been demonstrated to correlate with reduced viral titers [302, 303].

Verdinexor (KPT‐335) is a selective inhibitor of nuclear export that binds specifically to XPO1 and inhibits its function, and in vitro studies have revealed that verdinexor inhibits the replication of different RSV strains following prophylactic or therapeutic administration [304]. Verdinexor treatment of RSV‐infected A549 cells reduced the expression of proinflammatory cytokines, which has important implications for its efficacy in vivo [304]. In addition, verdinexor showed antiviral activity against multiple influenza A and B viruses in mouse and ferret models [305]. A previous phase I clinical trial in healthy human volunteers demonstrated the safety profile of verdinexor, and its antiviral activity can be achieved at a tolerable dose range [306]. Verdinexor holds promise as a potential broad‐spectrum therapeutic agent for viral diseases.

### 4.5 Targeting M2‐1 Protein

The M2‐1 protein functions as an elongation factor during viral transcription, promoting the synthesis of full‐length mRNA [307]. This protein adopts a symmetric tetrameric conformation composed of three distinct domains: an N‐terminal CCCH zinc‐binding domain, a central oligomerization domain, and a core domain [308]. The zinc‐binding domain of M2‐1 regulates the processivity of the RSV polymerase and prevents premature transcription termination. This domain contains conserved amino acid residues—three cysteines and one histidine (Cys3–His1) motif—that coordinate Zn^2^⁺ binding. The zinc finger motif is essential for maintaining the protein's stable conformation and function [309, 310]. Previous studies have shown that the C7 residue within the zinc finger motif is critical for RNA binding and the formation of higher‐order complexes, and its mutation leads to a loss of M2‐1 function [79].

IBs are biomolecular condensates formed through liquid–liquid phase separation. During RSV infection, IBs form in the cytoplasm and play an essential role in viral RNA synthesis [311, 312]. IBs contain viral genomic RNA, the N, P, and L proteins required for replication and transcription, and also recruit host proteins such as mitochondrial antiviral signaling protein (MAVS) and MDA5, potentially modulating host immune responses by sequestering these factors [312, 313, 314, 315]. Earlier studies suggested that IBs contain subcompartments called IB‐associated granules (IBAGs), which specifically enrich newly synthesized viral mRNA and M2‐1 protein while excluding genomic RNA and core polymerase components (N, P, L proteins) [316]. However, recent findings indicate that M2‐1 does not directly participate in IBAG formation but is instead uniformly distributed within IBs or forms ring‐like structures [311]. M2‐1 maintains the transcriptional integrity of viral mRNA in IBs and, through interactions with the N and P proteins, indirectly supports the liquid‐like properties of IBs [311]. The critical role of M2‐1 in viral transcription highlights its potential as a target for small‐molecule therapeutics.

#### 4.5.1 Cyclopamine and A3E

The steroidal alkaloid cyclopamine (CPM) and its chemical analog A3E are condensate‐hardening drugs that effectively inhibit RSV replication [317, 318]. Studies demonstrate that CPM and A3E can modify IBs morphology, transforming them from functional liquid condensates into nonfunctional solid aggregates [317]. Drug resistance mutations reveal that A3E specifically binds to the R151 site of M2‐1 (a critical RNA‐binding residue), thereby disrupting its interactions with both viral mRNA and the P protein, ultimately compromising the stability of the transcriptional complex [318]. In mouse models, CPM and A3E exhibited significant dose‐dependent antiviral activity, effectively suppressing RSV replication in lungs and mitigating tissue inflammation [317]. This condensate‐targeting drug strategy may enable pharmacological modulation of previously undruggable targets, holding potential for developing broad‐spectrum therapeutics.

#### 4.5.2 AT‐2

AT‐2 (2,2′‐dithiodipyridine) is a zinc finger‐reactive compound that specifically targets the zinc finger motif of the M2‐1 protein [310]. Through covalent modification of cysteine residues within the zinc finger structure, AT‐2 induces Zn^2^⁺ release and subsequent protein conformational changes. In AT‐2‐treated RSV viral particles, M2‐1 protein forms intermolecular disulfide‐bonded dimers while its native intramolecular disulfide bonds are simultaneously disrupted [310]. These structural alterations render the M2‐1 protein nonfunctional, ultimately leading to viral inactivation. Notably, AT‐2 demonstrates potent virucidal activity, with complete RSV inactivation achieved after 24‐h treatment at 10 mM concentration [310]. The inactivated virus shows no detectable replication in cotton rat models. Importantly, AT‐2 inactivates the virus while preserving the surface antigen structure, thereby enhancing immunogenicity. However, this also increases the risk of vaccine‐enhanced disease, making it insufficient for creating a highly effective and safe whole‐virus vaccine against RSV disease.

### 4.6 Targeting NS1, NS2

Although NS1 and NS2 are nonstructural components of the virus and are only expressed within infected host cells, they are essential for enhancing viral virulence [319, 320]. NS1 consists of 139 amino acids, and its crystal structure reveals a β‐sheet sandwich flanked by three α‐helices [321]. NS1 shares structural similarity with the N‐terminal domain of the M protein but contains additional helical structures not present in the M protein. This unique structure helps modulate host responses, such as regulating host gene expression, suppressing IFN signaling, and promoting inflammatory responses [321]. NS1 can exist as a monomer, dimer, or oligomer depending on the cellular environment, and this dynamic oligomerization state may be linked to its functional diversity [322]. In contrast, NS2 is composed of 124 amino acids, and its structure has not yet been fully resolved. Additionally, it lacks significant sequence homology with other known proteins [319].

NS1 and NS2 primarily inhibit the host's innate immune response, particularly the IFN signaling pathway [323]. During infection, NS1 and NS2 localize in the cytoplasm and exert their effects through the mitochondrial pathway [324]. NS1 can directly bind to MAVS, interfering with its interaction with RIG‐I and thereby blocking downstream IFN signaling activation [324]. NS2, on the other hand, binds directly to RIG‐I, preventing its association with MAVS, and synergizes with NS1 to enhance IFN pathway suppression [325]. NS1 and NS2 can also form a large protein complex called the “NS‐degradasome” on mitochondria, which degrades multiple IFN signaling molecules, including TRAF3, TBK1, IRF3/7, and STAT2, likely through the ubiquitin‐proteasome pathway [326]. This degradation mechanism provides the virus with an effective means of evading immune surveillance. NS1 also functions in the nucleus. Chromatin immunoprecipitation studies indicate that NS1 can enter the host cell nucleus and bind to the promoter or enhancer regions of immune‐related genes, influencing the transcription of inflammatory factors and antiviral genes [327]. The C‐terminal helix of NS1 is critical for its nuclear function, as mutations in this region significantly impair its ability to regulate host gene expression [327].

NS1 and NS2 also impact the adaptive immune response of the host. NS1 can inhibit dendritic cell maturation and disrupt immune synapse formation, thereby reducing T‐cell activation [321, 328]. It suppresses the proliferation of CD8+ T cells and Th17 cells while promoting Th2 cell activation, leading to a Th2‐biased immune response that exacerbates RSV‐associated inflammation [329]. Additionally, NS1 and NS2 activate the PI3K/AKT signaling pathway, upregulating NF‐κB‐dependent prosurvival genes and delaying host cell apoptosis [55].

The development of attenuated RSV vaccines lacking NS1/NS2 appears promising, with one advantage being their ability to promote IFN‐α/β expression, thereby inducing stronger innate and adaptive immune responses [320, 330]. Currently, no inhibitors targeting RSV NS1 and NS2 have been reported, but inhibitors against influenza virus NS1 have been identified. This is a single‐stranded DNA aptamer specific to the NS1 protein of influenza A virus, which can inhibit NS1 function to induce IFN‐β, and suppress viral replication without affecting cell viability [331]. This provides an important reference for the design of drugs targeting RSV NS1/NS2.

### 4.7 Targeting Host

RSV relies on multiple host factors for entry and replication while evading host immune defenses to complete its life cycle [332]. Targeting host factors is a promising antiviral strategy because it reduces the likelihood of RSV escape mutants emerging. However, the main challenge of this approach is avoiding undesired off‐target effects or toxicity. Numerous host factors have been shown to associate with RSV entry, including cellular receptors, coreceptors, and cofactors, such as CX3CR1 [67], HSPG [68], TLR4 [333], ICAM‐1 [334], annexin II [335], epidermal growth factor receptor [336], nucleolin [66], and insulin‐like growth factor 1 receptor (IGF1R) [337]. Resveratrol is widely recognized for its various therapeutic properties such as anti‐inflammatory, antiviral, and anticancer properties [338]. Notably, it could effectively block RSV attachment by binding to HSPG [339]. Moreover, resveratrol improved lung pathology in RSV‐infected mice, further confirming its anti‐RSV potential [339]. Recently, Ruan et al. found that tannic acid and daptomycin could combat RSV infection by targeting IGF1R [340]. Daptomycin, a circular lipopeptide antibiotic and an inhibitor of Zika virus infection [341], has also been found to reduce RSV viral load, inflammation, and airway resistance and protected alveolar integrity in RSV infection model [342].

In addition to these receptors necessary for RSV invasion, various host restriction factors can block viral infection by interfering with virtually every step of the viral life cycle [332]. Guanylate binding protein 5 (GBP5) belongs to a subfamily of IFN‐inducible GTPases involved in many important cellular processes, including inflammasome activation, signal transduction, vesicle trafficking, and innate immunity [343]. GBP5 targets the SH channel protein of RSV, efficiently inhibiting RSV replication. Moreover, GBP5 enhanced the IFN‐γ response, which has a strong antiviral effect on RSV infection [344]. Chemokine ligand 4 (CXCL4), a member of the CXC chemokine subfamily, is protective against RSV infection by blocking viral attachment through binding to the HSPG receptor, and the concentration of CXCL4 in plasma and airways correlates with the patient's viral load and the severity of disease [332, 345]. In vivo, CXCL4 treatment attenuated pulmonary inflammation and viral replication in RSV‐infected mice [345]. In addition, the ribosomal protein L13a, the IFN‐inducible proteins IFITM, IFI44, and IFI44L have been identified as host restriction factors in RSV infection, providing new avenues for innovative drug targets against RSV infection [332].

## 5 Current RSV Immunization Strategy

Significant progress has been made in the prevention of RSV, with approved mAbs and RSV vaccines now available for different populations. For infants, RSV prevention primarily relies on passive immunization and maternal antibody transfer. Due to its limitations, palivizumab is now reserved only as an alternative when nirsevimab is unavailable, specifically for high‐risk infants during the RSV season, with concurrent use of both mAbs prohibited within the same season [346]. Nirsevimab, as a long‐acting mAb, is recommended for all healthy infants during their first RSV season. For high‐risk infants (e.g., preterm neonates or those with CHD), a second dose may be administered at 8–19 months of age to protect against a second seasonal infection. However, it is contraindicated in infants with severe hypersensitivity to mAb components [346, 347]. As an alternative, Advisory Committee on Immunization Practices (ACIP) and Centers for Disease Control and Prevention (CDC) recommend maternal vaccination with Abrysvo at 32–36 weeks of gestation, which provides passive immunity to newborns through placental antibody transfer, though it is contraindicated in individuals allergic to vaccine components [348]. ACIP emphasizes that most infants only require one preventive strategy (either maternal vaccination or mAb immunization) and discourages simultaneous use of both [348].

For older adults, RSV prevention relies on active immunization. According to the June 2024 ACIP meeting, adults ≥75 years should universally receive Arexvy or mRESVIA vaccines, while those aged 60–74 years should be vaccinated based on risk assessment for conditions such as chronic obstructive pulmonary disease or congestive heart failure [13]. Additionally, ACIP acknowledges that RSV vaccination may benefit high‐risk adults aged 50–59 years, though no formal recommendation has been issued pending further clinical data [349]. All RSV vaccines are contraindicated in individuals with severe allergies to vaccine components, and administration should be deferred during acute febrile illness. Given age‐related immune decline, ACIP highlights that immunocompromised older adults may experience shorter antibody durability and advises completing vaccination before the RSV season, with coadministration of influenza vaccines to improve coverage [13].

However, current immunization strategies still have certain limitations. For maternal vaccination, although it can confer RSV immunity to pregnant women and passively transfer antibodies to the fetus for protection, this approach is subject to certain limitations [350]. Specifically, individual variations among pregnant women may affect antibody production and transplacental transfer, consequently influencing fetal passive immunization. Moreover, maternal antibodies typically provide protection for only the first 6 months after birth, with even more limited efficacy in preterm infants [351, 352]. Therefore, high‐risk infants should still be considered for nirsevimab supplementation even if the mothers have been vaccinated [348]. For acute RSV infection, the primary goals are to control viral replication, alleviate clinical symptoms, and interrupt transmission chains. Vaccines are limited in their ability to rapidly take effect postinfection due to the delay of specific immune responses, while antibody therapies, though capable of neutralizing free viruses, are ineffective against intracellular viruses and demonstrate limited efficacy during late‐stage infection or when viral loads are high. In contrast, antiviral therapeutics can directly inhibit viral replication and are effective at all stages of acute infection, making them particularly suitable for high‐risk groups with severe disease potential and immunocompromised individuals. Therefore, a combined strategy of seasonal vaccination and early antiviral treatment should be adopted for RSV high‐risk populations to minimize severe outcomes and exacerbation of underlying conditions. For immunocompromised patients with suboptimal vaccine responses, antiviral agents serve as an indispensable treatment option.

Most RSV‐related deaths occur in infants under 3 months of age and those in LMICs, so the accessibility of preventive measures in these settings must be prioritized. Each area should prioritize the most cost‐effective strategies based on local epidemiological data and work to integrate RSV prevention into existing immunization programs. More importantly, international organizations must drive global cooperation and resource sharing to reduce the economic burden of RSV mAbs in LMICs while ensuring prioritized access [14, 168].

## 6 Conclusions

Recent in‐depth studies on the structure, life cycle, pathogenic mechanisms, and host responses of RSV have paved the way for developing novel RSV therapeutics. Currently approved RSV vaccines, Arexvy and mRESVIA, demonstrate high protective efficacy in older adults, with even a single dose effective against RSV‐associated LRTD across three RSV seasons [353]. Meanwhile, the mAb nirsevimab is primarily used for passive immunoprophylaxis in infants. The advantage of nirsevimab lies in that it can provide lasting protection for up to 5–6 months through a single intramuscular injection, fully covering the entire RSV epidemic season. This is attributed to its modified YTE mutation which significantly prolongs the half‐life [154]. It exhibits a favorable safety profile, with injection site reactions and mild fever being the main adverse effects, both occurring at low frequencies [162, 354]. The World Health Organization has listed nirsevimab as a priority‐recommended product, and many countries have incorporated it into routine immunization programs. However, antibody therapies show limited efficacy in treating acute infections. Furthermore, most currently developed antibodies target the F protein, yet a single amino acid mutation in this protein can lead to the emergence of viral escape variants, resulting in reduced or completely abolished neutralizing activity—as demonstrated by the failed development of suptavumab.

Among the currently developed fusion inhibitors, although they have demonstrated antiviral activity in vitro and in early clinical trials, the clinical efficacy varies significantly across different populations, especially limited performance in severe cases and immunocompromised individuals was found. Notably, Ark Biosciences’ fusion inhibitor AK‐0529 has completed phase III trials in RSV‐infected infants, demonstrating promising clinical outcomes [245]. These agents generally have a good safety profile, with mild gastrointestinal reactions (oral formulations) or respiratory irritation (inhaled formulations) being the most common side effects. Drug resistance remains a potential concern, with resistance profiling of the fusion inhibitor presatovir identifying 16 mutation sites that reduce RSV susceptibility to the drug [355].

For polymerase inhibitors, representative drugs such as ALS‐8176 and PC786 have demonstrated potent inhibitory activity in vitro [258, 264], while inconsistent results were observed from clinical trials. In human challenge trials with healthy adult volunteers, oral ALS‐8176 demonstrated dose‐dependent reductions in viral load and symptom alleviation. However, when administered to hospitalized infant populations, the phase Ib clinical trial failed to replicate these therapeutic effects and instead revealed dose‐related neutropenia, which likely attributed to immature immune systems or distinct pharmacokinetic profiles in this pediatric cohort [260]. Similarly, the inhaled inhibitor PC786 showed symptom improvement in early trials, but its further development is currently on hold [267]. Additionally, the high mutability of viral polymerases poses a significant resistance risk for this drug class.

Therefore, despite belonging to different drug classes, all these therapeutics must address the challenge of resistance development. Error‐prone RNA polymerases, rapid and high‐level viral replication, viral fitness, and genetic barriers all contribute to antiviral resistance, thereby evading potential antiviral drugs or vaccines [153]. A promising strategy is the utilization of a combination of multitarget or multisite antivirals with synergistic effects [356, 357]. This strategy not only enhances the overall antiviral efficacy but also delays or prevents the development of resistance. Studies on humoral immunity have demonstrated that RSV G‐specific immunity plays a critical role in early disease control and viral clearance [10]. Compared with F protein‐targeting antibodies, G protein‐directed therapies can more directly modulate immune pathological responses induced by viral infection, particularly alleviating Th2‐type immune response‐associated symptoms. Combining G protein and F protein targeting strategies through bispecific antibodies may produce synergistic effects, offering more comprehensive protection. Additionally, combination of fusion protein inhibitors and polymerase inhibitors may serve as another choice, and in vitro studies have observed additive or mildly synergistic effects [357]. Another viable strategy is targeting host factors essential for the viral life cycle. Since human genes mutate at a much lower rate than viral genes, drugs directed against host proteins inherently possess resistance‐resistant advantages. However, host proteins are often involved in various physiological processes such as metabolic regulation and immune response, and drug intervention may lead to off‐target effects. Therefore, such drugs require careful evaluation of their clinical therapeutic window to ensure treatment safety. Furthermore, the global establishment of real‐time RSV surveillance networks and enhanced international data sharing represents a critical strategy for collectively addressing antimicrobial resistance challenges.

In addition to the issue of drug resistance, antiviral drug development faces some other challenges: (i) *Inadequate research models*. Current cell and animal models fail to accurately replicate human RSV infection characteristics, particularly postinfection immune responses and respiratory tract pathology, leading to discrepancies between promising preclinical results and clinical trial outcomes. The development of humanized animal models or human‐derived organoids to study virus–host interactions may improve translational potential. (ii) *Imbalanced drug target research*. The majority of current therapies focus on the F protein, with insufficient research on other vital targets like G and NS1/2 proteins that are crucial for viral pathogenesis. The scientific community should promote multi‐target coordinated development strategy and accelerate the development of novel inhibitor. (iii) *Clinical development difficulties*. Infants and young children constitute the highest‐risk population for RSV infection. Given their immature physiological and immune systems, clinical drug trials in this population present heightened safety concerns and ethical complexities. Adaptive trial designs and international multicenter collaborations are needed to accelerate research while ensuring participant safety. (iv) *Drug safety concerns*. Several investigational antiviral agents may induce certain adverse effects, necessitating particularly cautious evaluation in preterm infants or immunocompromised pediatric patients. Strategies such as chemical structure optimization and global adverse event monitoring systems could enhance safety.

In summary, the approved vaccines and mAbs have already covered the majority of high‐risk groups for RSV infection, showing promising potential to achieve RSV prevention across all age groups. Several small‐molecule drugs targeting key stages of the viral life cycle have entered clinical validation, and host‐targeted therapies provide new strategies to address viral resistance. The introduction of emerging technology platforms is particularly crucial—mRNA vaccine technology, gene‐editing tools, and AI‐assisted drug screening and design are accelerating the discovery and optimization of candidate drugs. Meanwhile, global research institutions and regulatory authorities should enhance collaboration by establishing an open‐access global data platform to facilitate real‐time exchange of clinical data and research findings, thereby accelerating research progress [358].

## Author Contributions

Y.Y., L.Y., and J.Y. contributed to the conception of the review. X.L., Y.Yin, Y.L., S.C., and Q.Q. collected the information and wrote the manuscript. Y.Y. and L.Y. contributed to the constructive discussions. All authors have read and approved the article.

## Ethics Statement

The authors have nothing to report.

## Conflicts of Interest

The authors declare no conflicts of interest.

## Data Availability

The authors have nothing to report.
